# High Throughput Extraction of Plant, Marine and Fungal Specimens for Preservation of Biologically Active Molecules

**DOI:** 10.3390/molecules15074526

**Published:** 2010-06-24

**Authors:** Thomas G. McCloud

**Affiliations:** Natural Products Support Group, Applied/Developmental Research Support Program, SAIC-Frederick, Inc., NCI-Frederick, Frederick, MD 21702, USA; E-Mail: tm200p@nih.gov; Tel.: +1-301-8467277; Fax: +1-301-8465206.

**Keywords:** natural products extracts, NCI-Frederick Screening Library, drug discovery, bioactive molecules

## Abstract

The Developmental Therapeutics Program (DTP) of the U.S. National Cancer Institute (NCI), at its NCI-Frederick facility, has built perhaps the largest and most diverse natural products screening library in the world for drug discovery. Composed of plant, marine organism and microbial extracts, it currently contains in excess of 230,000 unique materials. From the inception of this program to identify new anticancer chemotherapeutics from natural products sources in 1987, two extracts have been sequentially prepared from each specimen: one produced by organic solvent extraction, which yields a complex material that contains non- to moderately polar small molecules, and a water-soluble extract, a milieu largely unexplored for useful drugs in earlier years, which contains polar small to medium-sized molecules. Plant specimens and microbial ferments are extracted by modified traditional methods, while the method developed to produce extracts from marine organisms is unique and very different from that used by marine natural products chemists previously, but again yields both an organic solvent soluble and a water soluble material for inclusion into the screening library. Details of high throughput extract production for preservation of biologically active molecules are presented.

## 1. Introduction

**Figure 1:**
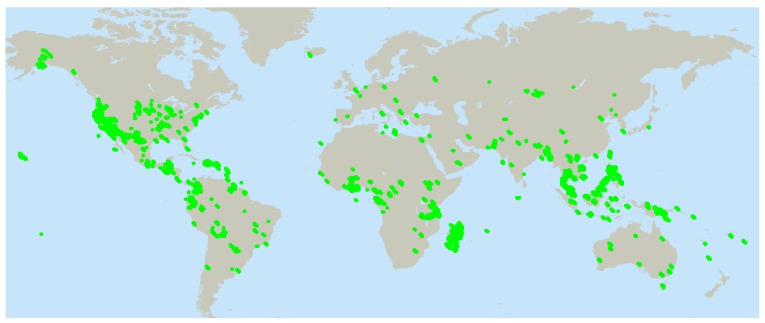
Plant Collection Locations (>60,000 data points).

Natural products continue to be a major source of pharmaceuticals and for the discovery of new molecular structures [1]. Having a large and diverse screening library of natural products available for rapid evaluation as new bioassays for newly-discovered anticancer targets become available is essential for this drug discovery process. The Developmental Therapeutics Program of the National Cancer Institute, USA, established the 60 human tumor cell screening panel for anticancer drug discovery in 1986. A component for drug discovery was establishment of a natural products library of extracts to be tested in this new discovery tool. In addition to screening for anticancer activity, this screening library has been used, and continues to be used, for many other purposes, including antiviral and antimicrobial drug discovery, chemotaxonomy and obtaining scarce pure substances for biological evaluation as potential drugs. This article, written from the perspective of a review of 20+ years’ experience in the production of these extracts from all sorts of specimens, contains the details of specimen processing and extract production. Approximately 65,000 plant specimens (Figure 1), representing >4,800 genera, and >19,700 species have been processed to yield >130,000 plant crude extracts by the protocol given below. Likewise, 15,000 marine specimens in >1,600 genera and >6,100 species of marine animals, >450 genera and >1,200 species of marine plants, and 20,000 fungal cultures in >2,100 genera, >5,000 species have been processed. 

**Figure 2:**
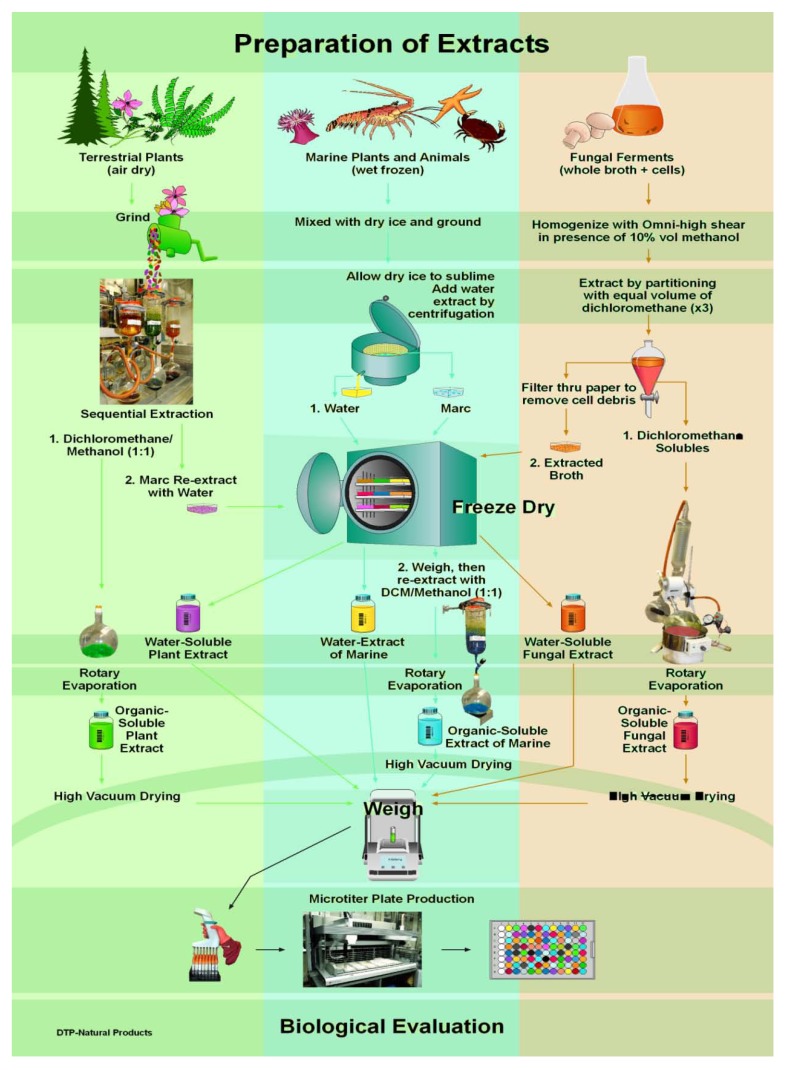
Flow Scheme for Production of Higher Plant, Marine Organism, and Microbial Extracts for Biological Testing at NCI-Frederick.

Figure 2, below, presents a visual shorthand for preparation of extracts as carried out at NCI-Frederick. Though the phrase ‘extracts obtained from the NCI Natural Products Repository’, or similar, has often been utilized by authors of scientific publications [2] when disclosing bioactive molecules that have been isolated from extracts obtained from this repository, details of the processing methods used to produce these extracts have not previously been published. Discovery of new chemotherapeutics is the goal, not the construction of a large library of natural products extracts. Yet to achieve this goal those bioactive substances contained in plants, marine invertebrates and microbial cultures must be extracted, concentrated, and preserved during storage without being altered through the processes. Only then are they present in the chemically complex matrix of an extract and available for detection by bioassay. We believe that the processing methods utilized in this program have been demonstrated to be effective, and to illustrate this, give a brief review listing examples of bioactive substances identified from these NCI-Frederick Repository extracts at the conclusion.

## 2. Plant Collecting

Plant specimens were obtained by the Developmental Therapeutics Program (DTP), NCI, NIH, through competitive contracts with botanical gardens possessing expertise in the flora of various regions of the world. Plant collecting began in January 1987 and continues today. All plant collections have been made in accordance with the NCI Letter of Collection, which protects the rights of the source countries, and which is published at the NCI Technology Transfer Center web site: http://ttc.nci.nih.gov/forms/loc.doc/.

Collectors are responsible for obtaining all applicable local permits, for taxonomic identification and documentation of samples, and for preparation and deposition of herbarium specimens to the Smithsonian Institution and/or at the other participating organizations: New York Botanical Garden for South and Central American plants (http://www.nybg.org/), as well as from some Caribbean countries, including Puerto Rico; Missouri Botanical Garden for African and Madagascan plants (http://www.mobot.org/); and the University of Illinois at Chicago (http://tigger.uic.edu/htbin/codewrap/bin/pharmacy/cgi-bin/depts/Medicinal_Chemistry_and_Pharmacognosy/index.php) for southeast Asian plants. Collections were later expanded to the continental United States and Hawaii, and U. S. territories such as Guam, through contracts with the Morton Arboretum (http://www.mortonarb.org/) and World Botanical Associates (http://www.worldbotanical.com/). Detailed collection records including latitude and longitude of collection as determined by GPS coordinates, date of collection, folkloric uses when available, and other taxonomic information are permanently stored in the Natural Products Repository Support System database. Figure 1 presents a visual depiction of the scope of NCI plant collection effort.

An early emphasis was placed on the collection of higher plants from rapidly dwindling tropical rain forest ecosystems. The importance of this will be made clear from the examples (*i.e.* calanolide and michellamine) given in Section 9 of this paper. Collections were made essentially at random, though with a preference for plant families known to produce ‘interesting chemistries’ such as the alkaloid-rich Rubiaceae and Apocynaceae, while plant families with little history for production of chemically or biologically interesting compounds, *i.e.* Poaceae, were largely discounted. Very small or endangered species and obviously diseased or damaged plants were explicitly not to be collected under the terms of the contracts. The specimens were field-identified, with herbarium specimens made, the bulk material was rapidly dried, sometimes with gentle warming, by the botanist-collectors, tagged with barcode labels and bagged for shipment to the US. Thick, woody materials were often cut or split in the field to promote rapid drying. Terms of the collection contract specified that samples of 500–1,000 g dry weight would be provided to the NCI. This specification favored collection of larger and longer-lived plants, such as woody perennials, which are easily obtained in quantity, over smaller or less common species. Sometimes the collector segregated leaf, seed, bark and root, *etc.* from a single plant into several specimens. There is a history of certain phytochemicals being localized in a single plant part [e.g. podophyllotoxin (CAS 518-28-5) from root of *Podophyllum peltatum*], which justifies this practice. And indeed, as the high throughput biological testing results of these ‘sibling extracts’ became available, there has been added value in determining that more than one extract from different parts of a single plant had shown a ‘positive’ bioassay.

## 3. Plant Grinding

High throughput grinding and extraction was a requirement of the new Anticancer Drug Discovery Program that was established in 1987, as the extracts which were produced were to serve as the ‘feedstock’ for the new 60 human cancer cell line panel which was to serve as the primary discovery tool used for detection of anticancer lead compounds [3,4]. Processing protocols had to be developed which were capable of producing many thousands of extracts from thousands of dry plant collections per year. But the goal was not simply to produce thousands of extracts, or to maximize the weight of extract obtained in the extraction process, but rather to produce crude extracts containing a representation of all molecules found in the specimen in their unchanged, un-degraded, and hopefully, biologically active state. Certain factors are widely believed to be enemies of the preservation of biological activity, including heat, chemical reactivity, time in solution, light, oxygen, *etc*. Consideration was therefore given to grinding methods, temperature exposure, solvents used for extraction and time in contact with solvent, solvent removal, and how materials would be stored when dry to achieve this goal. Following a period of methods development studies, which will be presented first, a generic processing protocol designed to achieve high throughput grinding and extraction with preservation of biological activity was developed, which is reported in detail here. 

**Figure 3:**
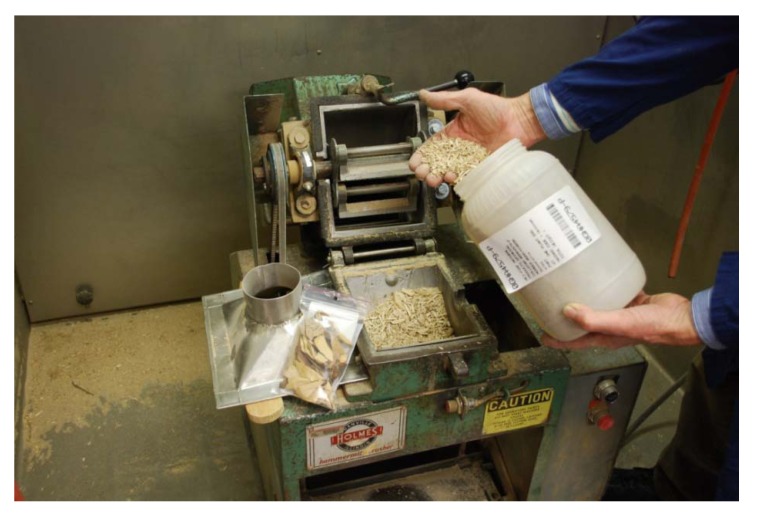
Dry Plant Grinding with a Holmes Hammermill. Note voucher, purpose-built stainless steel funnel, and barcode labeling.

In grinding operations, care must always be taken to avoid plant dust cross-contamination of other specimens, as well as protecting the technician from possible irritants and other deleterious effects of plant dust. Gloves, lab coat or other protective clothing, earplugs, dust mask and safety glasses are worn. The grinding mill is placed inside a 4 foot deep chemical fume hood (Figure 3) which has >1,000 linear feet per minute exhaust with the sashes pulled nearly closed, so that the dust which will be generated while grinding is taking place is vigorously pulled away from the technician and captured in a particulates filter. Specimen-contaminated materials including the bags which contained plants, contaminated protective equipment, floor sweepings, contents of vacuum cleaners and dirty particulate filters are incinerated on site. 

Since most plants acquired for this screening program are imported, this required that the facility obtain a United States Department of Agriculture (USDA) foreign plant importation permit. Typically the USDA insists that dried, imported plants be fumigated: this cannot be done in a drug discovery situation as a toxic residue from pesticides would be present in the extracts which are produced, and will kill the cells used to test for anticancer activity. Therefore, sealed boxes of dried plants as prepared by the collectors are moved through the import stations and while still sealed, moved into storage in -20 ºC freezers at NCI-Frederick. Specimens are stored at -20 ºC often for months until processed. The un-opened cloth bags containing the dried plant specimens are typically be removed from freezers and lay on the bench in the grinding area for 1-2 days to come to room temperature prior to grinding. To assure that no foreign plant diseases or pests could escape into the local environment, this grinding facility has been inspected and approved by the USDA. 

Exposure to heat is believed to be detrimental to preservation of biological activity. Heat is always generated in the grinding process. Milling dry plant specimens into extremely fine particles is detrimental in two respects: first - it takes extra time inside the mill, and as a consequence increases exposure to heat. And secondly, solvent extraction is a diffusion process, and is most effective and occurs most quickly when the particle size of the material being extracted is small. Yet if the particle size is extremely small, gravity flow of solvent through a glass column percolator is very slow, reducing the rate at which an extraction can be completed, clearly a negative factor to high throughput extraction. In order to achieve a high specimen grinding and extraction throughput, compromises must be made in grinding, particle size and completeness of extraction. Therefore, the protocol that was developed to achieve high throughput extraction is a compromise in which plant particle size distribution following grinding yields a powder with 80% of the specimen in the 0.5–2 mm particle size range. This compromise has benefit by limiting grinding time and exposure to heat, and in providing a material with good flow-through properties. 

A number of different mills were tested for their ability to grind the wide variety of plant materials that exist, their ease of operation and cleaning, and to produce the desired particle size range. There are many types of grinding mills, (knife or Wiley-type, impact, pin, *etc*.) which could be used to grind dried plants, but there is no single type which is ideal for all materials. Our experience has been that a hammer mill (model 201XL, Holmes Bros., Danville, IL, USA, http://www.holmesbrosinc.com) has been the most generally useful for the sort of work described here, being capable of pulverizing 3-centimeter-cubes of the hardest heartwood, nut or root, in addition to powdering leaves, twigs, and bark. As is shown in the photograph, (Figure 3), this mill is easily opened for cleaning. An infrequent drawback is that fibrous material will wrap around the rotor. A custom-fabricated stainless steel funnel (shown on the left side in Figure 3) directs the comminuted plant directly into a 4,000 mL plastic wide-mouth bottle, thus saving a transfer step from the metal ‘bin’ that comes with the mill. The choice of a suitable grinding mill is also dependent on the size of the specimens being ground, with micro-mills unsuitable for kilogram specimens, and larger mills unsuitable for specimens of a few grams. An unacceptable amount of sample loss will occur when small specimens are ground in unsuitably large mills. Other mills, used when needed, include the Wiley knife mill, Fitzpatrick high speed impact mill, Hobart hamburger grinder and Glen Mills hammer-cutter type V with top feed. An example of the appropriate use of this last item was the grinding of ~1,400 individual leaf collections for quantification of an alkaloid [5]. Mills are equipped with the largest motor available, so they can run longer without overheating, and pulverize larger pieces of plant without jamming. 

Before grinding, the specimen is examined to determine if it is dry enough to be ground, if it is properly labeled, and if there is anything unusual about it (such as fungus growing on it) that requires special treatment. If drying is needed the specimen goes into a vacuum chamber without heating. Attention is given to any HAZARD information that might have been included by the collector, such as THORNS, IRRITANT or ALLERGEN, with appropriate precautions taken (*i.e.* leather gloves, sleeve protectors, respirator) when handling the specimen. 

Only non-toxic lubricant such as cooking oil is used on mills to avoid contamination of a specimen with potentially toxic hydrocarbon grease. During grinding in any mill, care must be taken to avoid the introduction of other potentially contaminating materials such as paper (inks, glues, sulfites), cloth or non-plant foreign materials, which will later get into an extract. 

Prior to grinding, the identifying number placed on the specimen by the collector is read into the Natural Products Support Group (NPSG) local area computer network, thus creating an ‘inventory’ of what is in this lab, and which documents the processing steps. This software, which is based on dBase III+, has been made available to NCI collaborators as ‘The NCI Field Taxonomy Program’, is user configurable, and can be run on a PC. Plant taxonomy, grinding and extraction steps and barcoding are supported in this software.

A small sample of each plant specimen is not ground, but is saved as a ‘grind voucher’, which is selected to contain all the different components seen in the specimen (*i.e.* leaf, twig, bark), even obvious ‘contaminants’ (*i.e.* moss growing on bark) in approximately the same ratio as is seen in the bulk of the specimen. These vouchers, have been useful for comparison with the conventional taxonomic voucher or for chemotaxonomy, and are stored at room temperature in the NCI-Frederick Natural Products Repository. 

Reducing the size of plant pieces as provided by the collectors may be needed before they can easily be fed into the mill. This may include breaking twigs by hand, using a limb lopper to cut branches into smaller lengths, or use of a band saw to reduce hard woods and roots to sizes appropriate for the mill, typically under 3 cm. Cutting fibrous stems and bark across the fiber with the band saw is done to forestall a ‘ball of long fibers’ from accumulating around the armature of the hammer mill. The band saw is the most dangerous piece of apparatus in routine laboratory use, made more so by extremely hard and oddly-shaped roots which must be cut, and which sometimes contain embedded pebbles and even bits of metal.

The particle size obtained in grinding, and the residence time inside the hammer mill, is determined by the size of the holes in the outlet screen. Trials determined that for high throughput, minimum exposure to heating, and good rate of solvent flow-through during extraction, a 3/16" (4.7 mm hole diameter) mesh size installed in a Holmes 201XL hammer mill provided relatively small particles, most less than 2 mm, but not so fine that the flow rate of solvent through the sample, when packed in a columnar percolator, was unacceptably slow. A random sampling of plants and individual plant parts were ground with the 4.7 mm screen and other sizes. Then, utilizing a Tyler Industrial Products model RX24 portable shaker, the ground plant sample was sieved through a series of mesh sizes to obtain information about particle size distribution. Following vigorous agitation, the contents of each sieve was weighed. Table 1 shows an example representative of results obtained with a 4.7 mm screen, expressed as the percent falling into each size range.

**Table 1:** Particle size distribution for nine plant samples, expressed as a percentage.

**Plant Part**	**Size (mm)**				
	10 – 2.8	2.8 - 1	1 – 0.5	0.5 – 0.18	< 0.18
leaf/stem	11	**57**	21	7	2
root	12	**38**	24	14	9
root	44	**30**	13	6	4
bark	24	**39**	20	10	6
bark	28	**37**	15	14	6
wood	38	**46**	8	5	2
wood	50	**30**	9	7	3
fruit	43	**20**	16	12	8
fruit	30	**37**	17	12	5

These data document that when the 4.7 mm screen is used in the hammer mill, ~70% of the resultant ground product is smaller than 2.8 mm diameter, while an excessive quantity of fines <0.18 mm is not produced. Grinding time for a typical 1 kg plant specimen is 5 minutes, with roughly twice that spent in cleaning the mill prior to the next specimen. Between each grinding, the mill is opened, cleaned first with a vacuum cleaner, followed by a cloth towel moistened with ethanol or isopropanol, and finally with a blast of compressed air to remove any residual fine dust or solvent. An occasional specimen will leave a ‘waxy’ residue in the mill, which is most easily removed by passing corn cobs through the operating grinder. The ground weight of each specimen is recorded to the database. This weight is important to know so that the percent yield of any molecule of interest that is obtained from this specimen can be calculated. Should gram quantities of a biologically active molecule be required in the future for more comprehensive biological evaluation, it is important to be able to inform the botanist whether recollection of one or one hundred kilos of the plant is needed to obtain the required quantity of compound. Yield of a compound from its source should always be given in any publication, and recording a ground weight, which in our situation is also the weight extracted, allows this calculation to be made.

## 4. Plant Extraction: A Sequential Two-Step Process

### 4.1. First Step: Extraction with Organic Solvent

Plants contain a great variety of chemicals, so there is no single solvent that will solubilize all of them. From initiation of the Natural Products Drug Discovery effort in 1987, it was decided that a water-soluble extract, as well as the traditional organic solvent extract, would be produced and tested for every specimen. 

Methods development trials were carried out using dried plant specimens of over a dozen different species known to contain biologically active substances covering a broad range of polarities. Pure solvent as well as organic solvent mixtures were compared for their ability to extract these substances from ground plants by several techniques, with extracts bioassayed (Table 2). Not tried as an extraction solvent was dimethylsulfoxide (DMSO), which, though this solvent is often used to dissolve extracts and pure chemicals for biological screening, has many problems, not the least of which is a high boiling point of 189 ºC, making it impossible to remove at a low temperature, and thus impractical for large scale extraction. 

Some well-known techniques were also evaluated for purposes of comparison, though they were considered totally impractical for high throughput extraction at the numbers and mass required by this NCI program. Soxhlet extraction was never seriously considered, primarily because of the potentially negative consequences of long-term boiling in organic solvent of materials being prepared for biological evaluation. Methanolysis and other unpredictable and irreproducible chemistries could occur during such treatment of extract. Supercritical fluid extraction (SFE) was unsuitable for our purposes for several reasons including the size and expense of the extractors that would be required and the volume of CO_2 _that would be used. A frequently-cited benefit of SFE extraction is its ‘environmental friendliness’ in not using organic solvents, yet if the desire is to extract the full range of polar substances from the natural matrix of plant it is necessary to utilize an appreciable quantity of an organic solvent modifier, such as 30% methanol, when performing SFE. Clearly, the environmentally friendly aspect is lost when use of appreciable organic solvent plus release of CO_2_ to the environment are considered.

**Figure 4:**
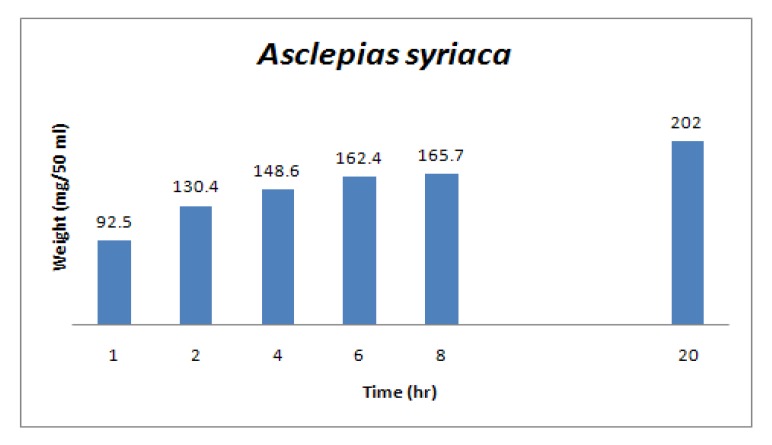
Increase in weight of organic solvent extractables as a function of contact time.

Solvent extraction is a diffusion process. To extract small molecules from an insoluble matrix, solvent must first penetrate the matrix, mobilize the substance, and only then can the substance diffuse from the matrix and into the surrounding extraction solvent. This takes time - but an optimum time for contact with extraction solvent was not known. In addition, the mass of materials extracted from a specimen will be somewhat dependent on the number of times the substrate is extracted. During methods development studies a number of plants were extracted for varying periods of time, and with repetitive passes of solvent, with the weight of extracted material determined at each step. If the goal is to achieve high throughput extraction with preservation of biologically active molecules, time is an enemy on both counts. As is seen in the representative example presented as Figure 4, extraction of small, organic solvent soluble compounds has already reached ~50% (wt/wt) in the first half hour. But double the yield will be obtained if steeping in organic solvent is allowed to continue for ~20 h. 

In the final protocol, partly for these reasons and partly to establish a reasonable schedule for the technicians who perform the work, plants are typically placed into percolators and immersed in solvent during the afternoon on one day, they soak overnight, and are then drained the next morning, so the specimen typically spends ~15 h in contact with organic solvent. During non-working contact hours the percolators, with lids on, hang inside chemical fume hoods at room temperature in the dark. 

**Figure 5:**
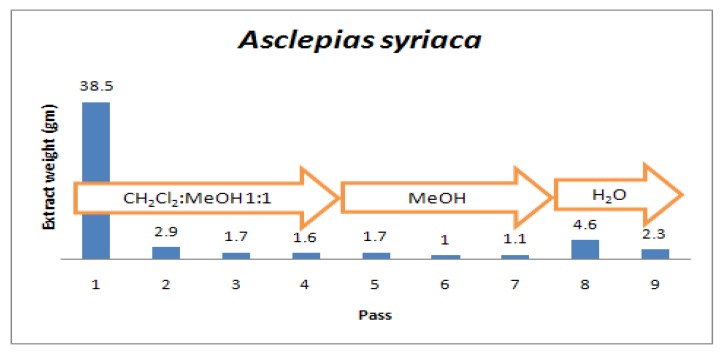
Extractables as a function of the number of solvent passes through a percolator.

It would seem obvious that the greater the number of passes of solvent through the plant marc, *i.e.* the residue following extraction in the percolator, the greater will be the weight of the extractables obtained. But to achieve high throughput extraction, a balance must be reached between maximizing the mass yield *versus* the cost of solvents and time spent in performing multiple extractions. In Figure 5 is shown a representative example of a series of studies that were done, which demonstrate that following ~15 h of contact time, the vast majority of organic solvent soluble material is typically contained in the first pass of 1:1 dichloromethane (DCM)/methanol (MeOH). Each additional pass follows ~15–24 h of contact time. Three additional passes with DCM/MeOH gives only a total 14% increase in mass, with an additional three passes with 100% MeOH increasing the yield by only another 7%. It is judged not worthwhile to expend the additional time and solvent (DCM is an expensive solvent) to obtain only a 14% increase in extract weight. The increase in extract mass at pass 8 by water clearly indicates that polar substances which are not appreciably MeOH soluble are being mobilized. 

An alternative protocol would have been to place the ground plant material in a suitably large container, such as a 6 L borosilicate Erlenmeyer flask, cover with solvent, stir, shake or sonicate, and filter. The composition of this organic solvent extract is much the same as that of the first pass of solvent through a percolator, but there is some evidence that this technique will produce a lower yield of extractables in a single pass, and re-extraction and cleaning of apparatus will be more time-consuming and labor-intensive than alternative methods. 

Following solvent removal, each extract from this methods development study was bioassayed by the methods used to search for anticancer substances, primarily toxicity to human cancer cells grown in tissue culture. The solvents and protocols used to make extracts of aliquots of a single plant specimen, *Asimina triloba* (Annonaceae), known to contain bioactive substances, are shown in Table 2. This is a representative example of the many trials that were performed. In this case the known bioactive substance is a linear acetogenin, asimicin, (CAS 102989-24-2), [6], (one of many congeners known from this plant, which also produces alkaloids), a very nonpolar molecule. The mass extracted is greatest with the DCM/MeOH solvent mixture when compared with the other organic solvents that were used. DCM/MeOH efficiently dissolves the acetogenins from the plant matrix, as is shown by the high toxicity of the organic solvent extract. For comparison, examine the data for water extractables: though the mass extracted by water is substantial, water is found to be a poor solvent for solubilization of asimicin, as is demonstrated by the lack of toxicity of this water extract. 

**Table 2:** Bioactivities obtained from extracts when produced by various solvents and in different orders. [EtOH is lab grade un-denatured 95% EtOH. P388 is a mouse leukemia cell line. BSL is lethality to brine shrimp (*Artemia salina*) carried out as described by Meyer, *et al*. [7], expressed in mg/mL. Other cell lines are human-cancer derived, members of the NCI 60 cell line panel [3], with toxicity expressed as µg/mL.] * indicates re-extraction of a marc.

Asimina triloba Annonaceae	EtOH	MeOH	MeOH/ Toluene	MeOH/ CH_2_Cl_2_ 1:1	*MeOH rinse	*Aq reextract	H_2_O/ MeOH 9:1	*MeOH/ CH_2_Cl_2_ reextract	H_2_O
Ws,Sb,Tw,Lf									
**% Extractable **	**1.7**	**2.6**	**2.4 **	**4.2 **		**2.7 **	**2.4 **	**1.8 **	**3.3 **
BSL (mg/ml)	0.01	0.01	0.08	0.02		1.0	0.28	0.02	0.24
P388 (in vitro)	0.26	0.46	0.48	1.8					
A549 Asc-1 (Lung)	<0.24	<0.76	<0.67	<0.75		>362.0	7.6	<0.9	39.0
HT29 (Colon)	<0.24	<0.76	<0.67	<0.75		>362.0	182	<0.9	61.0
SNB-19 (CNS)	<0.24	<0.76	1.1	0.8		>362.0	58	<0.9	38.0
UO-31 (Renal)	56	62	0.8	4.5		>362.0	29	16	19.0
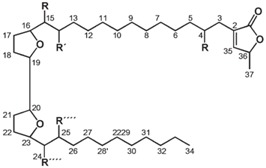									

95% ethanol, being readily available and cheap in most localities, has an historical record of being used as a solvent for extraction of plants. In the case presented above, as was found to be typical for other plants tried, it is seen that while the 95% ethanol extract is biologically active, the mass of extract obtained from the plant specimen is the lowest of those organic solvents tried, while the DCM/MeOH solvent combination extracts more than twice the mass. 

**Table 3:** Some Methods Development Plants and Bioactive Compounds Contained Therein.

Genus/species	Plant Part	Common Name	Bioactive Contained	Chemotype
*Brassica oleracea *	Leaf	cabbage	nontoxic control	glucosinolates
*Asimina triloba *	WS/SB/TW/LF	pawpaw	asimicin	acetogenins & alkaloids
*Podophyllum peltatum *	Root	mayapple	podophyllotoxin	lignins
*Trewia nudiflora *	Seed		trewasine	macrolide
*Camptotheca acuminata *	Root	happy tree	camptothecin	alkaloid
*Taxus brevifolia *	Bark	Pacific yew	taxol & other taxanes	alkaloid
*Cephalotaxus harringtonii*	Stembark		harringtonine and congeners	alkaloid
*Gossypium hirsutum *	Seed	cottonseed	gossypol and many others	terpenoid
*Asclepias syriaca *	ST/LF/SD	milkweed	cardiac glycosides	
*Castena dentata *	TW/LF/BK	chestnut	tannins	polyphenolic

During these methods development trials observations were made regarding handling of plant materials with widely different physical characteristics, and how these affected grinding, extraction, solvent removal and drying. Specimens which are very fibrous or contain an abundance of lipidaceous materials can present problems in grinding, while highly colored extracts may later present problems in color-sensitive bioassays. Nonetheless, from these trials, there was no case found in which a known biologically-active substance was destroyed by any of the grinding, extraction, and processing methods that were tried. But there definitely are differences in extract quantity and composition depending on extraction solvent used and order of extraction: certain known non-polar toxic molecules were not found in the water extract when water extraction was performed first, and certain highly polar substances were not detected in the organic solvent extract when only non-polar organic solvent extraction was performed first. In addition to order-of-extraction, *i.e.* organic solvent first or water first, a variety of pure organic solvents and solvent combinations were explored for their effectiveness in freeing biologically active substances from ground plants. When both weight of crude extract as a percentage of initial dry plant weight and biological activity were considered together, a protocol in which the ground dry plant is first soaked in a 1:1 DCM/MeOH solvent mixture overnight, followed by a 100% MeOH wash of the plant marc, with those two combined to give the crude organic solvent extract, gave the best yield of bioactive extract of any protocol evaluated. It is our belief that the use of the relatively non-polar solvent DCM, (P’ = 3.1) to mobilize and remove the waxes, lipids and essential oils so frequently found in plants renders the more polar molecules in that plant material more accessible to subsequent dissolution by MeOH and later, water. For dried higher plant specimens, a protocol in which the organic solvent extraction is performed prior to water extraction is the most economical of time and resources, and most compatible with high throughput extraction and screening. 

At a later time, and with the increasing emphasis on removal of halogenated solvents from the workplace, extensive empirical trials lead to the development of the 6:3:1 MeOH/ethyl acetate/methyl tertiary butyl ether mixture as a solvent suitable for re-dissolution of the organic solvent extracts which had been obtained through DCM/MeOH extraction. Though not tested as exhaustively as the other solvents and mixtures given in Table 2, some trials with this solvent for plant extraction indicated it was comparable to the DCM/MeOH solvent system in effectiveness. 

**Figure 6:**
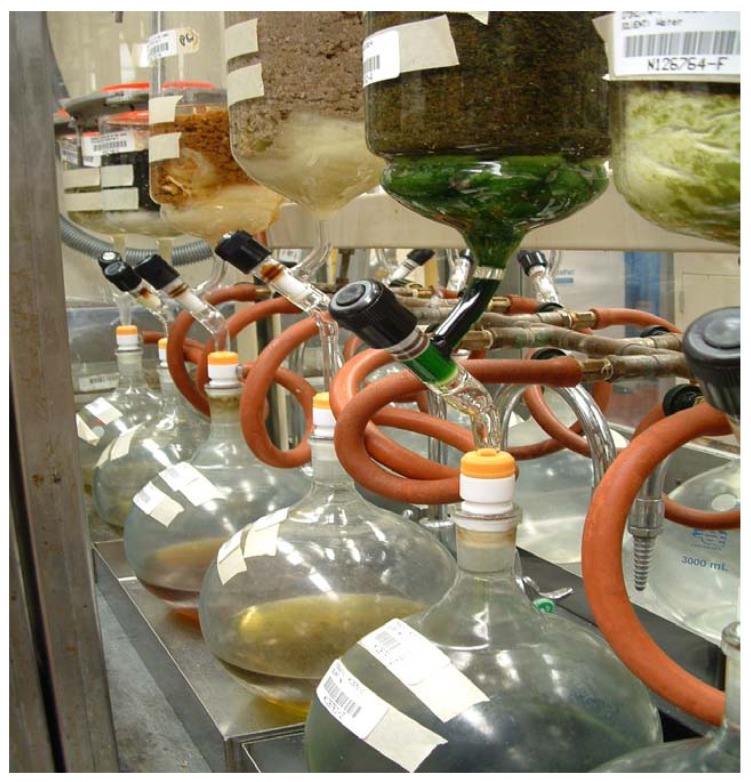
Percolators in use, illustrating bar-encoded labeling over masking tape, cotton filter plug, high vacuum Teflon petcock, vacuum adapter, and 3 L plastic coated flat bottom receiver flask.

A glass percolator of the appropriate size and configuration for our needs was not available for purchase in 1987, so following trial and error experimentation, a design was drawn and fabricated from heavy walled borosilicate glass tubing to our specifications. Percolator/extractors of this design are now commercially available from Kontes Glass, (http://208.72.236.210/html/pg-584800-Flasks.html, # 584800). These percolators are 10 cm in diameter and of various lengths to give volumes of 1, 2, 3, 4 or 6 liters, and are fitted with a Teflon-plug high vacuum petcock. Though the Teflon petcock is expensive, the big advantage it brings is that it does not leak, as the standard thru-plug petcocks always do. By insisting that petcocks, percolator diameters, percolator stems, *etc*. be all of uniform size, these components become interchangeable, so a plug does not have to be kept with a specific valve, all perk-hangers are of a single diameter, and all vacuum adapters fit all stems. Eight of these percolators, each attached to a 3 L receiver flask with necessary accessories fit inside a 6 foot chemical fume hood. Having the floor of the hood raised from the floor of the room by 18 in. puts the percolators at a convenient height (Figure 6). 

A small plug of cotton is placed in the stem above the Teflon valve to trap fine particulates, preventing them from entering the valve body, and a pad of cotton is placed across the inside base of the percolator to aid in rapid draining of solvent (Figure 6). A percolator that is 25% larger than the total volume of ground plant material must be selected as the individual receptacle for each specimen, as dried plant biomass swells upon solvation. Light, fluffy plant material can be compressed into the percolator to conserve space and solvent. A vacuum adapter is placed on the stem leading into a 3 L plastic coated, flat bottom flask (Figure 6). 

An appropriate barcoded label, which contains information identifying both plant specimen and extract, is placed on the percolator and the flask. Since the protocol produces two extracts from each specimen, it has proven quite useful to use identifying numbers for organic solvent extracts that always have an odd number as the final number, and conversely, an even number is always the last number of a water soluble extract. Barcoding is used to track all specimens, extracts and fractions during lab processing. Non-contact laser readers are placed in numerous lab locations. Barcoding saves considerable time that would be spent in writing labels and records, and eliminates transcription errors. An extremely tough label is required, one which remains readable despite exposure to organic solvents, abrasion, transfers, heat and extreme cold, water, high vacuum, *etc*. We have had favorable experience with Kimdura 96 labelstock, adhesive 091, and a thermal transfer printer, such as the SATO M84 Pro. This labelstock will stick to nearly any surface to which it is applied, glass or plastic, so long as the surface is dry, not oily and not exceedingly cold. Once applied, it cannot be peeled off without destroying the label. When transfer of a label is necessary, as it is in extraction lab operation, this problem is overcome by applying the labelstock to masking tape on the glass. The masking tape, with barcode label attached, can then be transferred from the glass percolator to plastic-coated round bottom flasks for rotary evaporation, and finally, the label peeled from the masking tape and applied to the plastic-coated 120 mL ‘bulk bottle’, the permanent repository storage vessel, permanently. Likewise, the masking tape with barcode attached is transferred to a RB flask containing water extract, then subsequently to a Pyrex dish or stainless steel tray for freezing and freeze drying. At the time the lyophilized extract is transferred into a ‘bulk bottle’, the barcoded label is peeled from the masking tape and applied to the bottle permanently. 

Analytical reagent grade DCM and MeOH, purchased in 200 L drums, are always used to make extracts. These are mixed in a 1:1 ratio in a flask and sufficient volume is poured over the plant in the percolator to completely cover the plant, plus about 5 additional centimeters, as dry plant material swells as it solvates. A cap is placed on the percolator (not in contact with the organic solvent) and the specimen allowed to steep overnight at room temperature, in the dark, inside the chemical fume hood. 

The protocol which was eventually adopted specifies soaking overnight in DCM/MeOH in a cylindrical percolator, the extract drained and the plant marc, untouched in the same percolator, covered in 100% MeOH, then soaked for an additional 30 min. This additional MeOH extract is drained into the same flask as the initial DCM/MeOH extract. These first and second passes are considered to be a single solvent extraction, and are assigned only a single extract number. Rapid draining of solvent is facilitated by attachment of a vacuum hose to the side-arm of the adapter which is between the flask and the perk (Figure 6). When gentle vacuum is applied, the solvent quickly drains from the perk. When organic solvent flow has slowed to a drip, the petcock is closed and 100% MeOH added. The re-extraction in MeOH serves a useful purpose aside from increasing the weight of the organic solvent extractables fraction. Since this plant marc will be extracted sequentially with water, and that water extract will be frozen and freeze dried, the re-extraction step in 100% MeOH serves to remove essentially all of the DCM from the marc prior to the addition of water. This is important since the low-boiling DCM is problematic inside a freeze drier, and would be problematic for disposal of a halogenated solvent containing waste material.

**Figure 7:**
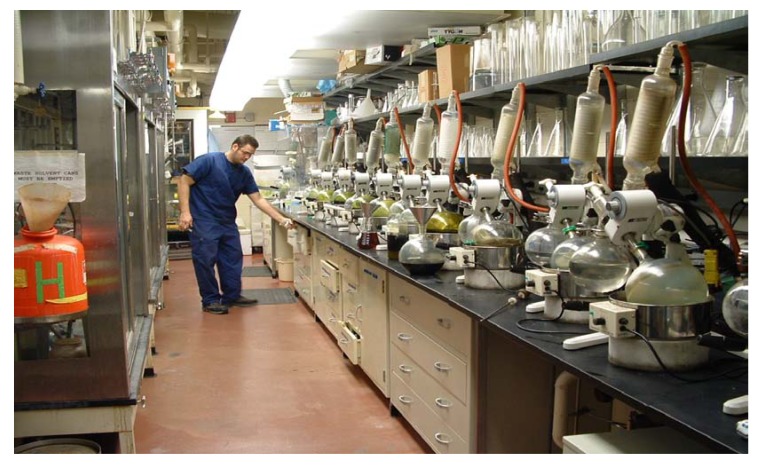
A 12-unit manifold of rotary evaporators. All glass is plastic safety coated. Note the stainless steel steam ducts in the rotovaps and stainless steel funnels.

**Figure 8:**
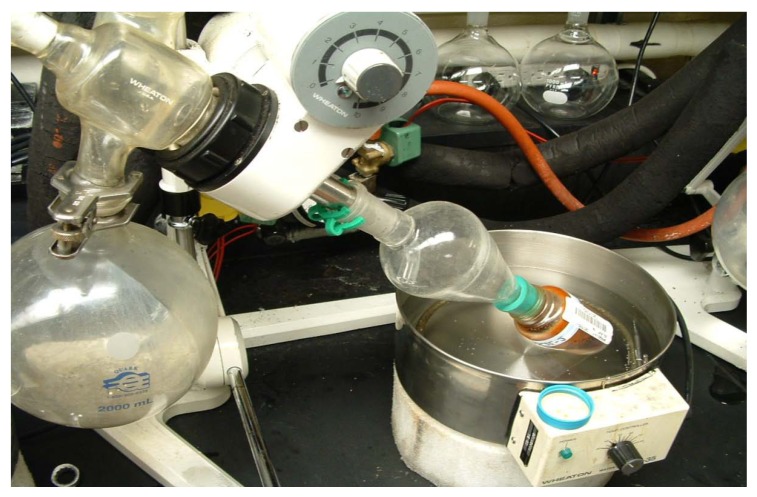
Rotary evaporating to concentrate extract into a bulk storage bottle. Note design of bump trap, Keck clip, Neogreen stopper, vacuum-rated solenoid tied to rotovap rheostat and multiple labeling of the bottle.

This combined organic solvent extract is moved to a rotary evaporator (‘rotovap’, or thin film evaporator, Tokyo Rikakikai, http://www.eyelausa.com/category.php?cID=1), where, with gentle warming from a waterbath (<40 ºC) and good vacuum, (with vacuum control decreasing from 300 mT to 20 mT as drying occurs) and a condenser chilled with recirculating water at near 0 ºC, the solvent is quickly (*i.e.* 1L/h) removed to yield a concentrated crude organic solvent extract of the plant. The rheostat which controls the speed of rotation of each rotary evaporator is wired with an electrical tie-in to a vacuum-rated solenoid, so that whenever the rotary evaporator is turned on, vacuum is immediately supplied, but more importantly, if turned off, the solenoid is closed so that flasks can be added and removed at will from a manifold of 12 rotary evaporators without losing vacuum to the whole manifold (loss of vacuum could result in flasks dropping into the water baths) while other rotovaps continue to operate. At each vacuum port is a needle valve which provides the technician a mechanism for individually restricting the vacuum of each rotovap, to minimize bubbling. The vacuum utility available in most laboratory buildings is generated by a liquid ring pump which produces an ultimate vacuum no better than 300 mbar, which did not meet our requirements. The quantity of water that would be consumed if water aspirators were used for vacuum generation in an operation of this scale eliminates this option from consideration. To operate 30^+ ^rotary evaporators every day at full production, a building utility 3-stage steam ejector vacuum generation system was installed (model MV41.1.80RHNZ - http://www.graham-mfg.com/index.asp?pageId=16), which produces <20 mbar at the benchtop, and provides more than adequate vacuum. The vacuum level on each of two manifolds of 12 rotary evaporators is regulated by a vacuum controller, with the Buchi 161 being particularly applicable because if its ease of use. Both vacuum level and hysteresis are easily adjustable. As the vacuum improves and the extracts become more concentrated, (*i.e.* less DCM, more MeOH/water/essential oils remain in the rotating flask), the setting is decreased to give a better vacuum, with faster removal of the higher boiling solvents. Neslab CFT-75 recirculating water chillers provide near 0 ºC water to the rotovap vertical condensers. Selection of vertical rather than horizontal condensers permitted 12 rotary evaporators to be installed on 27 feet of linear lab countertop space, (Figure 7). Another component of each vacuum manifold is a large, dry-ice filled, double-jacketed stainless steel can which serves to trap solvent vapors that escape condensation on the rotovap condensers. When the extract has been concentrated to a thick, yet pourable, viscosity it is transferred into a suitable container, and to maximize the amount of extract in each storage bottle, continued addition of small amounts of extract can be made when a suitable bump trap is used, and rotary evaporation continued (Figure 8). Notice this style of bump trap (Quark Glass http://quarkglass.thomasnet.com/item/all-categories/qa-34-adapter-distilling-trap/pn-6720?&plpver=10&origin=keyword&filter=&by=prod) has a tube bent at an angle to the steam duct. Having the large bulb of the bump trap in which bubbles can break and liquid drain back into the bottle, and the bent tube restricting direct upward flow, usually prevents bubbles of extract from getting into the condenser. A ‘soft seal’ is formed between the bottle and the bump trap with a ‘Neogreen’ rubber stopper with an appropriately sized hole bored through it. Organic solvent extracts that are drained from the percolators each morning are rotary evaporated, transferred into borosilicate storage bottles, and placed onto the high vacuum drying manifold for overnight drying within an 8 h work day. 

Because of an excessive breakage rate of glass ‘steam ducts’, those rotating tubes which carry the rotating flasks, virtually indestructible stainless steel steam ducts were manufactured for us at this facility. Likewise, stainless steel funnels are used in the extraction lab (Figure 7-Figure 8). 

At the time the concentrated extract is transferred into a bottle, the barcode label which identifies the extract is also transferred. The storage bottle also has a handwritten extract number plus the tare weight of the bottle in grams (to an accuracy of +/- 0.1 g) written on a tape label. If an extract is to be stored for a prolonged period, it is highly recommended that to minimize decomposition with loss of biologically active molecules, the permanent storage vessel be borosilicate glass, not alkaline soda glass. At NCI-Frederick the permanent repository storage vessel is a 120 mL wide mouth, plastic safety coated, borosilicate glass bottle (Wheaton Glass - special S-942R3 type I wide mouth packer) with a Teflon lined cap. As soon as bottles are removed from the rotary evaporators they are placed onto dry ice. The final step in extract preparation is to place each of these bottles into a vacuum bottle for overnight drying at a vacuum of <40 µ, to remove all traces of solvent or water. Then each bottle is weighed and that weight entered into the NPSG database. Though highly variable, it is common for 5% of the weight of the dry plant specimen to be soluble in organic solvent. Following removal of an aliquot for biological testing, the remainder goes into long term storage in -20 ºC freezers at the NCI Natural Products Repository in Frederick, MD. 

Waste organic solvent which has been trapped by rotary evaporator condensers and dry ice traps, consisting of DCM, MeOH, some volatile plant organics and water, is disposed of by a licensed hazardous halogenated waste contractor. No recycling or distillation is performed on-site.

### 4.2. Second Step: Extraction with Water

The plant marc remaining in the percolator, following draining of the organic solvent, is not entirely dry, since it still contains some residual MeOH. Experience has shown that having a small amount of MeOH present improves the efficiency and yield of the water re-extraction. However, excessive organic solvent will make the water extract impossible to freeze even at -40 ºC and will create a problem inside the freeze drier. Methanol at ~5 to 8% of the volume of the water used for re-extraction improves the yield, will freeze into an ice slush, and is manageable, if not ideal, inside a freeze drier. Therefore, after MeOH has been drained, but before water is added to the marc, a flask is placed underneath the percolator as before, so that vacuum can be applied. A slight vacuum is intermittently pulled until it is obvious that all dripping has stopped, and the plant marc has become partly dried. Then high purity water (Milli-Q >2 µohm resistivity, 0.2 µ final filter) is added until the marc is covered, and the Teflon petcock is closed. Some additional swelling of the plant marc is often seen. An appropriate barcode label is applied to the receiving flask, designating this as a water extract of a specimen. After the plant marc steeps in water overnight, it is drained the next morning by applying slight vacuum to the flask. This water extract is transferred, along with its companion barcode labels, into a tray of suitable size for freezing and lyophilization, and placed into a -40 ºC ‘sharp’ freezer (*i.e.* a freezer with hollow shelves through which Freon circulates so that samples will be frozen very quickly. Freezers which cool by circulating air over a heat exchanger are not suitable because of the requirement that the heat exchanger frequently pass through a defrost cycle). After the lyophilized product is transferred into a permanent storage bottle, this same label, now separated from the masking tape, is transferred onto the permanent storage bottle. When the volume of aqueous extract is 3 L or greater, stainless steel trays with 5 L capacity are used, but for smaller volumes, borosilicate glass (Pyrex) cake dishes with a flat bottom are used. Freezing and freeze drying of ~<50 mL aliquots of aqueous extract directly in storage bottles also is done. Since this extract contains some MeOH, even in a freezer set to -40 ºC the extract may well have a ‘slushy’ consistency. Placing ‘slushy’ extracts onto dry ice for two hours prior to loading into the freeze drier helps in preventing excessive bubbling inside the freeze drier as the vacuum improves. If the extract remains semi-liquid at -40 ºC, insufficient organic solvent had been removed from the plant marc prior to the addition of water. Dilution of slushy extracts with additional water, and re-freezing to produce a near-solid material is required to give a product acceptable for freeze drying. Rotary evaporation of aqueous extracts to remove the organic component is not done because of concerns about accelerated hydrolysis due to long-term heating. The spent marcs are dumped from the percolators and incinerated on site, with percolators washed in an industrial glassware washer through an alkali, acid, rinse, distilled rinse cycle. 

**Figure 9:**
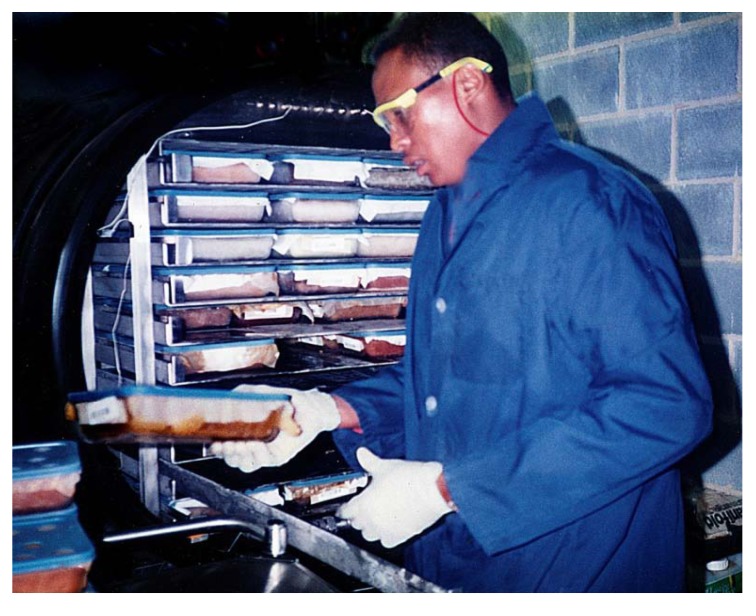
Pyrex dishes containing frozen plant aqueous extracts, capped with paper and a porous lid, are loaded into a VirTis SRC-250 freeze drier.

Just prior to loading into the freeze drier, a filter paper held in place by a perforated lid (Figure 9), is placed on each container of frozen extract so as to minimize the possibility for cross contamination due to ‘spitting’, which could occur inside the freeze drier. Capped dishes and trays are promptly transferred onto freeze drier shelves already cooled to -30 ºC, the door is closed and a drying cycle initiated. While maintaining a shelf temperature of -30 ºC or below, and watching carefully for foaming or bubbling (due to incomplete freezing or the presence of organic solvent), vacuum is pulled down to ~300 µ or less over the course of 1 h. It is sometimes necessary to freeze the materials even lower than -30 ºC, or maintain a vacuum of ~1 mT overnight to stop excessive foaming or bubbling. To optimize drying time, it is essential that a very good vacuum be attained prior to increasing the shelf temperature. Freeze drying when organic solvent is present in the aqueous extract is best accomplished in a machine which has a product chamber and remote condenser chamber. Over the course of the first ~48 h of ‘freeze drying’ the residual organic solvent in these aqueous extracts in the product-side of the machine is removed, essentially by ‘bulb-to-bulb’ distillation, and is remotely condensed on the condenser plates. The organic solvent has a higher partial pressure than water, so if present inside a freeze drier, decreases the ultimate vacuum that can be achieved, thus slowing the drying process. The deleterious effect of having liquid organic solvent in the condenser chamber is minimized by suspending the condensed organics inside the vacuum chamber so they are not in contact with the side walls. This is accomplished by installation of a funnel inside the condenser chamber that will collect drips from the condenser plates, and direct the liquid toward the drain. Much of the methanol-containing slush is then removed from the chamber by having two manually operated ball valves installed in series, with ~100 mL dead volume between them, plumbed onto the drain line. These are alternately opened/closed as required to remove the condensed organic solvent without breaking the system vacuum. After ~ 2 days of operation, the freeze drier compressor is briefly turned off and a very fast partial defrost performed on the condenser chamber to remove the ice, which still contains trapped organic solvent. Then the freeze drying process resumes, typically for 5 more days. These freeze driers are equipped with Alcatel 2063 pumps, the pump oil protected from organic solvent contamination with a liquid nitrogen trap. Even so, pump oil is changed after every second load. When maintained in this way, the pumps have performed nearly non-stop for in excess of 10 years. 

Nearly all of the >110,000 aqueous extracts produced in the NPSG extraction labs have gone through VirTis SRC-250 freeze driers, remote condenser machines which have ~5.6 square meters of temperature-controlled shelf space on the product side. A machine of this size will accommodate 140 different plant aqueous extracts of ~1,200 mL in Pyrex dishes per run cycle, or 30 of the stainless steel trays which are used when the volume exceeds 3 L per extract. For an ice cake of 3 cm thickness, 2 days at -30 to -20 ºC, followed by defrost, followed by 5 days at a shelf temperature near 0 ºC will produce a batch of completely dry extracts. Shelf temperature is not set higher than zero so that heat-sensitive molecules are preserved in their native state. In a high throughput extraction operation such as this the composition of product being loaded into the machine is different each time, so there can be no ‘fixed protocol’ for operation. A certain amount of adjustment of operating conditions is always needed to produce the best result. Though lyophilization of a thin ice cake could be carried out in less time, if the aqueous extract is quite dilute, the residual lyophilized powder will be very thin and quite difficult to efficiently scrape from the dish. Our choice has been to work with a thick ice cake, a greater number of extracts per run cycle, and a longer freeze drier run time. 

On the day that dry samples are scheduled for removal from the freeze drier, the shelf temperature is raised to ~+15 ºC for several hrs to raise the temperature of the now-dry extract to near room temperature so that condensation of humidity onto this hygroscopic powder will be minimal. Upon release of vacuum and opening of the door, the group of technicians works rapidly to scrape the dried extract from the dish and transfer it into a tared 120 mL borosilicate bottle, the goal being to get the bottle tightly capped before the sample adsorbs humidity from the air and becomes sticky. The physical properties of dry aqueous extracts are quite variable. Some are highly colored, others near colorless. Some are light and fluffy, others hard, and others have the consistency of taffy. From each lyophilization dish, the pre-printed barcoded label is transferred onto the bulk bottle, then each bottle is weighed with the weight recorded to the NPSG database. Though highly variable, it is common for an additional 2–4% of the weight of the ground plant specimen to obtained by water re-extract following an initial organic solvent extraction.

**Table 4:** Average percent weight extracted by organic solvent (DCM/MeOH), followed by water. (Relatively low numbers are the result of most specimens containing multiple plant parts.)

Plant Part	Number of Extracts	Weight percent extractable in organic solvent	Weight percent extractable in water (re-extract of marc)
fruit	>2,500	7.0	4.5
leaf	>10,500	6.8	3.9
twig	>5,000	3.4	2.2
stem	>5,500	2.9	1.8
bark	380	4.8	2.5
wood	>5,000	2.2	1.2
root	>5,500	3.9	2.1
Overall average yield	> 68,000	5.0	2.8

### 4.3. Discussion of Plant Extraction

The purpose of the plant marc re-extraction with MeOH is not to increase the amount of extract obtained, though it does that, but rather to remove DCM. The presence of residual DCM in the subsequent water extract is a significant problem in freezing and freeze drying, so removing as much DCM as possible is essential if re-extraction of the marc with water is planned. In addition to increasing the yield by re-extraction with MeOH, the removal of residual DCM from the marc by MeOH extraction produces an essentially halogenated-solvent-free waste material, *i.e.* the plant marc, which can be safely disposed of even if water re-extraction is not required. The 5% residual MeOH in the plant marc does not pose an insurmountable problem in freezing or freeze drying, but does serve as a co-solvent with water and increases the mass of water extractables. 

To measure the effectiveness of this protocol, a random series of plant marcs which had gone through the protocol as given above were transferred to a Soxhlet extractor, with extraction done by refluxing MeOH for five days. Though weight extracted is variable, a Soxhlet re-extraction in MeOH rarely delivered as much as 30% additional mass of organic solvent extractables. Thus, the protocol as outlined above typically removes >70% of the mass that would be obtained by a much lengthier, repetitive, more costly extraction process. Most clinically-used small molecule drugs would be found in a crude organic solvent extract if produced by the methodology given here.

Survival of microbes in DCM/MeOH for 15 h. was thought to be unlikely. To determine whether there were living microorganisms present in the aqueous extract, which is produced by steeping of the plant marc in water for ~15 h. at room temperature, a number of randomly selected aqueous extracts were streaked onto nutrient agar and incubated for several days. The colony count was quite low, and the organisms grew slowly, as if ‘weakened’. The microbes appeared to be of the common, airborne types, such as *Aspergillus* and *Penicillium*, which would normally be present in the lab air supply. No evidence was found that microbial action could be a significant factor in altering the composition of the water extract. 

During the peak period of extract production the lab was in operation continuously. Since the plant extraction protocol requires a two day period, plants were set up for extraction in organic solvent on Sunday through Thursday, with rotary evaporation taking place on Monday through Friday. On Saturday the aqueous extracts were drained and frozen, percolators dumped, washed and hung in the hoods, at the ready for resumption of this weekly schedule. With five technicians devoted to plant processing, 30 specimens per day were ground and extracted. Three freeze driers were constantly operating. In this way, 1,200 extracts each month were added to the screening library, and fed into the 60 cell anticancer screening program. A ‘critical mass’ of lab personnel and lab apparatus is needed to achieve these efficiencies. This was accomplished in ~1,600 sq. ft. of lab space, containing two hammer mills, 36 linear feet of chemical fume hood space for hanging of extraction percolators, and 30 rotary evaporators on the benchtops. 

## 5. Extraction of Marine Specimens

Marine specimens in this context consist of both marine invertebrate animals of all sorts and marine plants, which are processed by a common protocol designed to preserve the biologically active molecules they contain. For simplicity, all of these are termed ‘marines’ in this discussion. Marine specimens are purchased through the competitive contract process by the DTP, with the same provisions as given by the NCI Letter of Collection. Most have been obtained from the general region of the South Pacific, most have been collected by scuba diving, with samples quickly frozen following collection and air freighted to the NCI-Frederick Repository on dry ice, where they remain stored frozen at -20 ºC until processed. Many photographs taken underwater of specimens which have been brought into this program are published in “Tropical Pacific Invertebrates” [8]. 

A typical specimen wet-frozen weight is ~1 kg. These specimens are ground and, as with plants, two extracts are made, but in this case an aqueous-first extraction is done by centrifugation, followed by lyophilization of both the aqueous extract and the marine tissue marc. Subsequently the dry marc is re-extracted by the organic solvent mixture DCM/ MeOH (1:1). When wet frozen specimens are being extracted, carrying out the water extraction step first is most economical of resources. The method given below has also been used with wet, freshly frozen higher plants, and also works with these materials. Among many trials done during methods development studies, marine specimens were either lyophilized and subsequently finely ground, or else finely ground prior to lyophilization, and next extracted with an organic solvent. The grinding of lyophilized materials produced no discernable benefits, but does produce a considerable amount of potentially irritating dust in the grinding process. Performing an organic-solvent-first extraction of lyophilized materials likewise produced no discernable benefit, but the marc, when re-extracted with water, quite commonly solvates into a ‘gel’ of sorts, from which a water extract is difficult to obtain. In the context of preserving biologically active molecules it is believed that a speedy water-first extraction protocol, minimizing the time that the specimen is not frozen, is most likely to preserve sensitive, biologically-active molecules contained in the marine specimens. 

### 5.1. Marine Grinding Procedures

Some marine organisms contain highly irritating 'slimes' and silicate 'spicules', the skeleton of a sponge, which are like fiberglass, that are unpleasant to get on the skin or into the eyes. Safety glasses, gloves and a lab coat with long sleeves sealed at the cuff are standard lab safety equipment for grinding and processing marine specimens. 

Marine specimens have a reputation among marine natural products chemists for rapid degradation when not frozen. Since the primary goal of the program for which these extracts are being produced is to discover new drug molecules, anything which alters the chemical composition of the specimen in a destructive or unpredictable way is undesirable. A series of methods development studies were performed designed to find a method for processing marine specimens which minimized the time they were NOT frozen, thus minimizing any destructive, unpredictable, irreproducible changes to the constituents in a specimen that would result in the loss of biologically active molecules.

**Figure 10:**
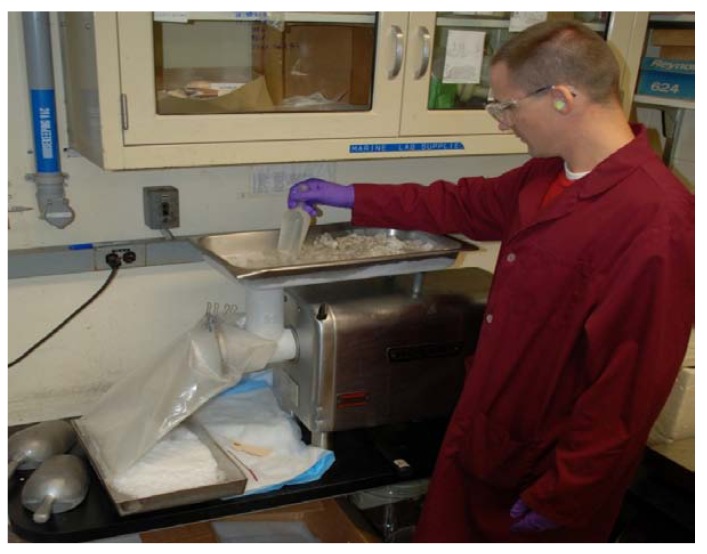
Grinding of marine organism tissue mixed with dry ice.

Specimens are stored at –20 ºC unless being handled. Upon beginning processing, a representative piece of the sample is taken and set aside as a voucher, along with all identifying labels (*i.e.* collectors dive tags). This ‘grind voucher’ is kept frozen until it is lyophilized. It is of use in marine taxonomy, *i.e.* for examination of spicule size and shape, or to perform chemotaxonomy by HPLC analysis of an extract made from a piece of the lyophilized voucher. The frozen bulk specimen, sometimes composed of several organisms of the same species and typically having a wet-frozen weight of ~1 kg, is initially broken into pieces smaller than approx. 1 inch cubes depending on hardness. To accomplish this the thick plastic bag containing the marine specimen is buried in dry ice for some time. Then, with a hammer and a chisel, the specimen, contained in a heavy stainless steel tray, is pounded until only small tissue pieces remain, though additional methods are sometimes needed (*i.e.* hydraulic press - particularly with hard corals or shells, or band saw). Next, removed from the plastic bag and mixed with dry ice pellets, sufficient time is allowed for the tissue to become very cold, and VERY BRITTLE!, as this is the key to good grinding. A Hobart hamburger grinder, the same as is used by a grocery store, (Hobart model 4822, with 3/8 inch (9 mm) faceplate and special hardened knives - 00-290372) is assembled and made ready to use by adding dry ice to the pan and grinding chamber so these are thoroughly chilled prior to addition of frozen marine specimen. A heavy duty plastic bag into which the ground specimen will drop, is attached to the front of the machine using a large clip. The base of the bag rests on a bed of dry ice (Figure 10).

With the grinder running, the frozen tissue pieces and dry ice pellets are SLOWLY! added, allowing the grinder plenty of time to do its’ work, while the technician listens for sounds of the motor straining. It will jam on occasion and it is imperative that the machine be turned off immediately to avoid overloading the circuitry and/or damaging the gearbox of the machine. The amount and speed of sample addition is dependent upon the specimens' characteristics and only operator experience will assure infrequent jams.

Approximately two times the volume of dry ice is added to the specimen to keep the mechanism and housing cold during grinding. There are a few marine animals such as starfish, sea squirts, holothurians, *etc*. that remain gummy even at dry ice temperature, and thus do not grind well. These are also broken into pieces while mixed with dry ice, but just prior to grinding, liquid nitrogen is poured over the combined dry ice pellets and tissue pieces. At liquid nitrogen temperature every marine specimen we have encountered, even holothurian, is rock-hard and can be ground. Pieces of specimen tissue are "chased" from the throat by adding more dry ice, and then the throat is taken apart and any remaining specimen added to the ground material in the bag. At this point the contents of the bag include the finely ground tissue of the marine specimen including shell if present, the ground saltwater ice which was a part of the specimen and powdered dry ice. A tag with appropriate identifying barcode label is attached to the bag with wire, and twisted securely. The bag containing this ground sample is placed inside a –20 ºC freezer, where it will stay for (usually) three days, during which time the dry ice sublimes. 

At completion of grinding of each specimen, the head is removed from the body of the machine, disassembled, cleaned thoroughly by placing in the sink, and immediately dried to avoid rust formation. When reassembling, all possible sticking points are sprayed with PAM corn oil or similar food grade lubricant. Petroleum oil is not used for lubrication because it would be toxic to cells in tissue culture, and should never be allowed to contaminate an extract. To avoid cross-contamination and hazards to technicians, the work area is thoroughly cleaned, with any potentially contaminated materials, plastic bags, boxes, gloves, dust masks, *etc*. disposed of, and subsequently incinerated. 

Marine invertebrates and wet-frozen marine plants, and wet-frozen higher plants from a saline environment (*i.e.* mangrove), wet frozen fungal basidiocarps and ‘waxy’ nuts/fruits have all been finely ground successfully by this protocol. Hard corals, bivalves, and other animals with hard shells are crushed with a hydraulic press prior to grinding, but the shell is passed through the Hobart grinder as well. A number of other grinding techniques (*i.e.* blenders, ‘squeezers’, impinging plates, *etc*.) were tried during methods development studies, with the hamburger grinder of the type mentioned above found to be most suitable for this grinding-while-frozen protocol. Though the grinder throat, perforated plates, augers and knives all wear out as a result of this very challenging and abrasive grinding, and must be periodically replaced, the motor and gearbox have survived 20 years of hard use.

### 5.2. Marine Aqueous Extraction by Centrifugation

A number of extraction methodologies for preparing water extracts were investigated during the methods development stage of this work, including dialysis, squeezing, and various devices containing membranes, but these all suffer from the length of time required to produce an aqueous extract. Since marine specimens have a reputation for deteriorating very quickly when they are not frozen, it is the goal to complete all processing steps that have to be done while the wet marine specimen is not frozen in LESS THAN 2 HOURS, from the time the material is removed from the –20 ºC freezer until the marc and water extracts are back into a freezer. Centrifugation is the best choice for high throughput production of aqueous extracts because of its high capacity and speed of extract production. 

It has been observed that if ground specimens are allowed to stay in the –20 ºC freezer for more than 5 days, some become ‘leathery’, so do not extract well by the basket centrifugation methods described here. Therefore, it is important that aqueous extraction of a ground marine specimen take place 3 to 4 days following the grinding, and after all the dry ice has sublimed from the specimen. The bag containing the ground specimen is removed from the freezer, its contents transferred into a 4 L beaker, and sufficient milli-Q high purity water is added to make an approximate 3:1 mixture. This is the first time the marine specimen has not been frozen since its collection. Mixing is done while inside a refrigerator (*i.e.* deli-box or, chromatography refrigerator) at ~4 ºC, through use of a mechanical device such as the Con Torque motorized paddle stirrer (Eberbach Corp. model 7225 fitted with a MF-53H, 5" D × 1/2" Bore, High Pitch type paddle) for approximately 45 min., until an homogenous, ice-free aqueous slurry has been achieved.

**Figure 11:**
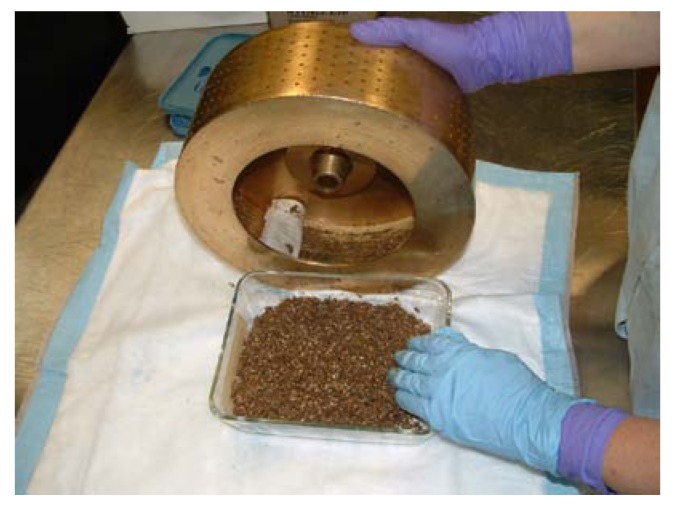
Basket rotor model 1357 with the insoluble marine tissue trapped by paper being removed and transferred into a borosilicate dish for freezing and lyophilization.

The centrifuge rotor (Damon/IEC model K centrifuge 7165 with perforated basket rotor model 1357) is prepared by lining the rotor with a strip of pre-cut Whatman 3 mm chromatography paper (*cat.* 3030-690). With the paper set firmly against the walls of the rotor and moistened with high purity water, it will adhere to the rotor wall with no bubbles or creases. With a 4 L flask in wet ice set to receive the filtrate coming through the draining hose from the rotor catch-basin, and with the rotor spinning at 1,200 to 1,500 rpm, the aqueous slurry is slowly added to the center of the basket rotor. The rotor speed may be increased to hasten filtering, but speeds above 2,000 rpm are rarely necessary and may compress the filter cake thus slowing the flow rate. NEVER! set rotor speed above 2,500 rpm with this type of rotor. Aqueous extracts, which are often 3 to 5 L for a 1 kg specimen, are poured into barcode-labeled stainless steel trays suitable for freezing and freeze drying. The filter paper with compressed tissue marc attached is removed from the rotor and placed in a properly labeled dish (Figure 11). Both the aqueous-soluble extract and marc plus paper are frozen in -40 ºC ‘sharp’ freezers, then lyophilized. Freeze drying of marine aqueous extracts and marcs typically takes five to seven days when the ice cake is 3 cm thick. Following freeze drying and transfer into tared borosilicate bottles, the aqueous extract is weighed and an aliquot taken for screening. The dry marc is also weighed, then re-extracted with DCM/MeOH to produce the organic solvent extract for screening. 

Various complications may occur while centrifuging, such as torn paper, clogged paper, and marc material "finding its way” into the receiving flask. Torn paper and marc is removed and placed into a labeled dish, then new paper is placed in the rotor and centrifugation is started again, passing the aqueous fraction which has been collected through the filter paper a second time. Scraping a clogged paper is often an effective way to restore the filtrate flow, but great care must be taken not to tear the paper in doing so. With experience one may be able to foresee a clogging problem from the consistency of the slurry (*i.e.* a difficult holothurian) and therefore try to forestall the problem with the use of a filter aid, (*i.e.* Celite). Placing a coarse pre-filter of fiberglass on top of the paper filter inside the basket rotor allows for filtration of some difficult, slimy materials. Occasionally nothing works, and an entire sample is frozen and lyophilized. In any case, all extra weights, *i.e.* paper, fiberglass, Celite, *etc*., which have been added to the marc, and will remain after lyophilization, are recorded.

These aqueous extracts contain salt, and that saltwater is very corrosive to metal parts, so thorough and frequent washing of apparatus is important. The basket rotor should be cleaned, taking care to remove residue from underneath the rim, and then dried carefully. The inside of the centrifuge is rinsed and wiped dry after each specimen has been done. All glassware is washed thoroughly promptly after emptying. All items are rinsed well with high purity water before being put away. 

If a single technician is devoted full time to the pounding, grinding and centrifuging portions of marine extraction, a production rate of 30 specimens each week is achievable. 

### 5.3. Organic Solvent Extraction of Marine Marcs

Lyophilized marine marcs are set up for extraction with organic solvent immediately upon removal from the freeze drier, or if that is not possible, then stored under vacuum. Marcs that contain spicules can be extreme skin irritants in much the same way as coarse fiberglass, and spicules are not the only potential irritants. Handling these marcs while wearing thick rubber gloves and a long sleeved lab coat with cuffs secured, and inside a hood, is standard procedure in this extraction lab and is strongly recommended to others who may be engaged in this sort of work. Despite all the processing that a marine specimen goes through, in one case a technician received an unpleasant sting on an un-gloved hand from a freeze dried Portuguese Man ‘O War marc which had gone through all of the above procedures! 

Although the wet-frozen weight of each specimen had already been recorded, in order to calculate the potential yield of a ‘molecule-of-interest’ obtained from one of these extracts, having the dry weights of both aqueous extract and marc is required. Therefore, the lyophilized marc in its vessel is placed on a balance and the gross weight is measured. Then the marc along with the filter paper is transferred into a percolator. (See plant extraction protocol for percolators and their preparation). The empty vessel is returned to the balance and its weight subtracted. The weight of the filter papers and/or filter aids is also subtracted from the gross weight to obtain the lyophilized weight of the marine tissue itself, which is recorded to the permanent database. If this weight and the weight of the lyophilized aqueous extract are added, an approximation of the weight that would have been obtained had the entire, original wet-frozen specimen been lyophilized can be calculated. From this, an approximate yield of a molecule of interest from either water extract or organic extract can be calculated, and an estimate of the yield per kg from the original collection may be obtained.

Inside the percolator, any sizable clumps of marc are broken into small pieces, and pressed down to minimize the volume of solvent required. Enough 1:1 DCM/MeOH is added to cover the marc with at least one inch of solvent. Marine marcs tend not to swell in organic solvent. After soaking overnight, the organic solvent is drained from the percolator, and the marc is covered again, this time with pure MeOH. After soaking another half-hour, the MeOH is drained, and the combined organic solvent extracts are rotary evaporated to give a concentrate. Organic solvent extracts of marine marcs generally dry quickly without foaming. The concentrate is transferred into an appropriate storage vessel, further dried under high vacuum, weighed and the dry weight recorded. Refer to the plant extraction protocol above for greater detail for these steps.

### 5.4. Discussion of Marine Extraction

When this protocol is followed, most of the ‘slimes’, high molecular weight materials (*i.e.* polysulfated polysaccharides [30] peptides and some proteins), and the sea salt that is present in the original specimen, are found in the aqueous extract, thus the weight of these lyophilized aqueous extracts is typically relatively high. A random selection of aqueous extracts were ashed at 500 ºC with the residue then weighed. The ash portion measured from 5% to >70% the weight of the lyophilized aqueous extract, and most commonly was >30% of the weight. Thus, a significant mass of each lyophilized aqueous marine extract is comprised of inorganic materials.

A random group of lyophilized marine aqueous extracts were resolubilized in water and passed through a 0.2 µ filter. Following drying and weighing, it was found that typically >95% of the aqueous extract had passed the filter. This assures good solubility of these extracts when prepared for bioassay.

Several marine natural products chemists who have looked at the data which has been accumulated over the years have questioned the low weight of DCM/MeOH extractables, 3–5% the weight of the specimen, which is typical of this method. It should be kept in mind that sea salts, which are soluble in MeOH to the extent of ~18 g/L, and other substances, are removed from the specimen by this water-first extraction protocol, prior to organic solvent extraction. Thus, the weight of organic solvent solubles obtained with this protocol is expected to be somewhat small when compared with other traditional marine organism extraction methods, such as steeping a specimen in MeOH or isopropanol. Since there are essentially no salts present in an organic solvent extract prepared by the method outlined above, these organic solvent extracts are relatively enriched in smaller, moderately polar and non-polar molecules compared to extracts prepared by traditional approaches. These small molecules are the ones most likely to have drug-like properties and in extracts prepared through this water-first method are not diluted by the presence of biologically inactive salts. Thus, when these extracts are placed into microtiter plates on a weight basis, *i.e.* 50 µg/well, the biological test system is exposed to a higher concentration of these small organic molecules than would be the case with extracts prepared by more typical methods. We believe this results in a greater likelihood that bioactivity will be detected in the marine organic solvent extracts prepared by this protocol. 

Extraction of marine organisms following this protocol has gone well, certainly from the technical standpoint, and with >15,000 successfully completed. As pointed out previously, by performing much of the process on frozen tissue, many of the commonly encountered difficulties associated with marine specimens are avoided. By doing most of the marine specimen processing work on frozen materials, the possibility for decomposition with loss of sensitive biologically active molecules is essentially eliminated. A secondary benefit is that the unpleasant odors, skin irritations, slimes and the like which are reported so commonly when working with marine specimens have been extremely rare in this lab. 

Marine taxonomy remains problematic, but the best estimate is that the screening library presently contains approx. 1,600 marine animal genera, 6,100 species, and 450 marine plant genera, 1,200 species. Of >13,000 marine animal specimens processed, >5,000 are from the family Porifera, indicative of the great diversity of sponges in the marine environment. 

**Table 5:** Overall Average Yields from Marine Specimens.

		% Weight of specimen as extract	
	Number	DCM/MeOH	Aqueous
Animals	>13,000	2.2	14.3
Plants	> 670	1.6	21.8

## 6. Fungal Extractions

The viable fungal culture collection in liquid nitrogen storage at NCI-Frederick consists of ~12,000 pure cultures obtained from many sources including ATCC, university researchers, public health departments, the USDA, and by primary isolation done as a component of this program. It is common that taxonomy is incomplete, but these cultures are fermented, extracted, and only when the extract shows interesting biological activity is a more complete taxonomic determination done. When a culture is selected for fermentation, it is typically revived on potato dextrose agar (PDA), checked microscopically for purity, inoculated into PDA liquid culture media, and this seed stock is inoculated into a larger volume of PDA plus two or three additional media, with two grown agitated and one stationary. Typically, the volume of each of these fermentation broths is ~1 L. It has been the philosophy in this program that fungi will be more likely to produce a more diverse mixture of secondary metabolites after they have gone into senescence, so in addition to visual observation, the media is monitored for exhaustion of sugars, with harvest not done until sometime after sugar has been depleted. Typical fermentations are for 1-2 weeks, but occasionally cultures have been grown for over a month. Upon harvest, 10% the volume of whole broth of MeOH is added and the entire culture is high-shear homogenized to disrupt fungal cells (Omni-International, www.omni-inc.com/omni-macro-homogenizer-p-31.html). To determine the effectiveness of this homogenization in disrupting fungal cells, the quantity of ergosterol that could be extracted from un-homogenized *versus* homogenized cells was determined. Fungal broths were first extracted with methyl *t*-butyl ether, then mechanically homogenized and re-extracted by the high throughput protocol. Organic solvent extractables were saponified, then ergosterol was quantified by HPLC analysis on a silica column, developed in 85:15 hexane/ethyl acetate, isocratic, with detection at 282 nm. A significant amount of ergosterol was measured from the homogenized cells only. 

Homogenized whole broth is transferred to a separatory funnel with an equal volume of DCM added. Following shaking and separation of layers, the DCM phase is withdrawn, the organic solvent removed by rotary evaporation, and the concentrate transferred by syringe, through a 0.45 µ membrane filter, into a tared, labeled vial. After high vacuum drying, the weight of organic extract is recorded. For a 1 L fermentation, ~100 mg of DCM extract is a typical weight obtained, yet this small quantity is adequate for carrying out dozens of bioassays. The extracted aqueous broth is passed through filter paper to remove cell debris, yielding a clarified broth which is transferred to a barcode labeled dish, frozen and lyophilized. This freeze dried aqueous-soluble material is weighed and added to the screening library. Thus in the case of fermentations as well, two extracts are bioassayed: an organic solvent soluble extract and a water soluble material. The NCI-Frederick screening library now contains extracts of >2,100 fungal genera and >5,000 fungal species, and since most were fermented in three different media, greater than 30,000 fungal extracts are present in the screening library. 

**Table 6:** Overall Average Weight of Extract (based on ~13,000 most frequently utilized media).

DCM/MeOH	0.24 g/L
Aqueous	11.7 g/L

## 7. Miscellaneous Extractions and Processes

Several hundreds of specimens do not neatly fit into any of the three above delineated major categories. Dried lichens have been extracted as if they are dry plants. Mature fungal basidiocarps, collected fresh and delivered wet frozen to the lab, have sometimes been freeze dried, then extracted with organic solvent much like a dried plant. Alternatively, these high water content specimens can be placed into a large spark-free Waring blender with 75% MeOH/25% DCM and thoroughly minced. The organic solvent extract is clarified by passing through filter paper, and the marc re-extracted in the same way with water. Extracts were made of several *Caenorhabditis elegans* strains by pelleting the nematodes in a centrifuge tube then homogenizing and adding organic solvent to create a two phase system. “Traditional medicines” of various sorts, often poorly-described mixtures of components, are frequently sent to the NCI, and forwarded to this program, by private individuals as well as organizations doing research on Oriental and African traditional medicines, often with a protocol for preparation and administration. These submissions have generally been split into two parts with half being processed as faithfully to the instructions provided as possible, while the other half is processed by NPSG standard methods. In these cases, four or more extracts are submitted to anticancer testing per specimen, with testing results provided to the submitter. Semi-hardened plant saps and exudates are not amenable to standard processes, and have essentially no water soluble components (excepting simple sugars) in them, but sometimes an organic solvent extract can be made by mechanical stirring in an appropriate organic solvent, often methyl t-butyl ether being the best. There continue to be new challenges even after 20^+ ^years, and improvisation, based on experience, is frequently required.

During the mid-1990’s when extraction of specimens was a high priority of the NCI, seven technicians working in ~3,800 sq. ft. of laboratory space were routinely producing > 10,000 crude natural products extracts each year for biological evaluation. 

Removal of dried organic solvent extract from lab glassware, even utilizing hot detergent water under pressure, has always been problematic. After each use, laboratory glassware is passed through an industrial glassware washer which begins its cycle with an alkaline detergent, then an acid detergent, then a rinse and a de-ionized water rinse. A universally satisfactory detergent has not been found, so inspection and hand-washing of glassware is a continuing requirement. Long-term use of a single detergent was observed to lead to a residue in the washer and presumably on glassware as well, so the best solution to the problem is to change brands and types of detergent every few months. Each detergent has seemed to remove the residue left by its predecessor. There have been instances of stainless steel and borosilicate ware being permanently stained by substances present in an extract. Borosilicate dishes used to freeze dry aqueous extracts were treated with a silanizing solution, which makes a noticeable difference in the ease of which dried extract can be scraped from the dish.

**Figure 12:**
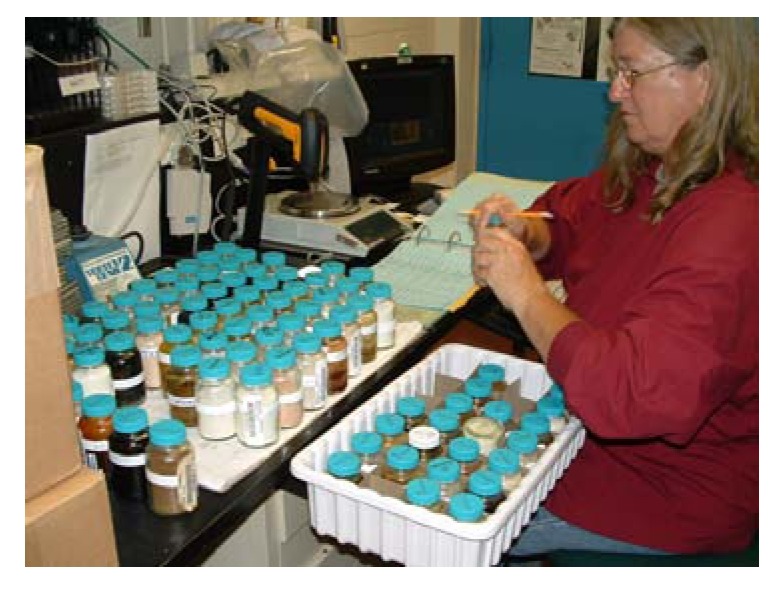
Recording weights of ‘bulk bottles’ containing vacuum dried extracts. Note multiple labels, non-contact laser barcode reader, and pan balance connected to computer work station.

With >230,000 extracts produced there has not been one totally lost because of a spill, breakage or accident (Figure 12). About a dozen 3L flasks with flattened bottoms have imploded while rotovaping, and in all cases the plastic safety coating contained both the glass and the extract. There have been several instances when biological test and retest did not correspond, so an examination of extract in comparison to grind voucher content was done by HPLC fingerprinting. In general, three extracts prior to and three past the extract in question were examined. There has been no occasion in which the composition of the extract differed significantly from the composition of the voucher. In laboratory operations as labor intensive as grinding and extraction are, there have unquestionably been some mistakes, but very few. Tracking through barcoding has helped greatly in minimizing mistakes through transcriptional error or mixing, keeping the identity of a specimen or extract at each step during processing beyond question.

The size of the specimens processed for this program has been relatively larger than those processed in most screening operations. The philosophy of this program from the start is that the initial specimen is large enough so that sufficient extract will be obtained to allow for the active substance to be isolated and its structure determined without a recollection of the source material. This has almost always been possible. The extraction protocols presented above were designed to be generic, high throughput, rapid, and to preserve those biologically active substances that may be contained, but these methods may not produce the highest yield of some substances. Once the structure of a compound of interest has been determined, customized extraction and processing methods have often been developed to optimize its yield. The same extraction laboratory personnel and equipment are used to perform up-scaled grinding, extraction by modified methods and large column chromatography (*i.e.* several kg of silica) to produce a semi-purified extract for delivery to the isolations chemist. 

## 8. Storage and Plate Making

At the end of 2009 more than 135,000 plant extracts, half organic solvent soluble, half water soluble, had been prepared by the protocols given here. Likewise, more than 29,800 marine invertebrate extracts, 4,200 marine plant extracts, and 36,000 fungal extracts had been produced by the methods given above.

From each 120 mL borosilicate glass “bulk bottle” an aliquot is weighed for production of microtiter plates. Aliquots in plastic tubes, each individually barcoded, are solubilized into an appropriate solvent, which is transferred by a TECAN robotic liquid handling system into multiple polypropylene 96 well, deep-well microtiter “Master Blocks”. When solvent is removed, each well contains a dry weight of 15 mg, which is an amount sufficient for hundreds of test plates to be produced for screening. Each of these “Master Blocks” is high vacuum dried and a heat-seal laminate applied prior to storage or shipment. 

At shipment, all data generated in the NPSG is uploaded to the Natural Products Repository Support System database. Extracts in borosilicate bottles, along with >170,000 microtiter plates containing aliquots of 88 different extracts per plate, reside in –20 ºC freezers at the NCI-Frederick Natural Products Repository in Frederick, MD, available for call-up. At over 230,000 unique extracts and still growing, this must certainly be among the largest and most diverse libraries of natural products crude extracts assembled for drug discovery anywhere in the world. Yet as large as it is, only ~10% of the known higher plant species are present, and lesser percentages of marine organisms and fungi. This library is considered a ‘National Resource’, available in both 96 and 384 well microtiter plate formats, ready for use not only by NCI labs at Frederick but elsewhere. Thus, the entire library can be screened on ~2,600 unique 96 well microtiter plates. Additional information for those interested in accessing the Natural Products Extract Screening Library can be found at http://dtp.nci.nih.gov/ branches/npb/open_repository.html. In addition to the initial screening in the DTP 60 human cell anticancer assay at NCI-Frederick, this library has been utilized in a number of diverse bioassays, such as antimicrobial, anti-HIV, and various whole-cell and molecular target assays. 

## 9. Bioactive Compounds from the NCI Natural Products Screening Library: “The Proof’s in the Pudding”

The creation of a large and diverse screening library is not an end in itself: the discovery of new, useful pharmaceuticals is the goal. At NCI-Frederick the primary anticancer screen is the 60 human tumor cell line panel, (http://dtp.nci.nih.gov/branches/btb/ivclsp.html), which detects cytotoxic and growth-inhibitory substances but additionally, provides information on selective cytotoxicity that, with the aid of the COMputerized PAttern REcognition algorithm (COMPARE), (http://dtp.nci.nih.gov/timeline/noflash/milestones/M7_COMPARE.htm), can be useful in predicting a mechanism of action for a compound, and in prioritizing those extracts for fractionation which exhibit a particularly unusual cytotoxicity pattern, perhaps indicative of a previously unexploited anticancer target. Nearly all of the 230,000 extracts produced in this program have been through this preliminary tissue-culture-based anticancer screening. A frequently asked, but ultimately meaningless, question has been: ‘What is your hit rate?’ The answer is entirely dependent on how you define a ‘hit’ and will be different for each individual assay. There are commonly interfering substances present in crude extracts, but these do not adversely affect all bioassays. Some bioassays are sensitive to tannins, others to lipids, others to highly colored substances. When 634 preferentially selected Euphorbiaceae crude organic solvent extracts were screened for PDBu displacement activity, 153 exhibited this property, so the hit rate was 24%, but from a very select ‘feed’ into the bioassay [14]. After >140,000 extracts had been screened for toxicity toward azole resistant *Candida albicans*, a ‘hit list’ of only 140 extracts was selected for dereplication, a 0.1% hit rate. But to make it onto the hit list, the extract had to show significantly lower toxicity toward bacteria and a human cell line being screened concomitantly. Thus, the emphasis is on selective toxicity toward fungi [21]. Simple toxicity in the 60 cell primary anticancer screen is not sufficient to make an extract interesting. From a subset of >19,000 crude plants extracts tested at 5 × 1 log doses in the 60 cell screen, 452 showed LC50’s below 50 µg/mL. But a highly toxic compound does not necessarily make a good clinically-useful chemotherapeutic. Only a few hundred extracts have shown patterns in 60 cell screening indicative of selective toxicity toward a tissue type. Fewer still have yielded novel bioactive molecules. 

With screening, as is always the case in natural products research, many known compounds, or homologues of known compounds, have been detected in these extracts, but often from new sources. This can be seen, for example, in a perusal of the listing of more than 400 compounds identified by the Molecular Targets Development Program at NCI-Frederick, https://ccrod.cancer.gov/confluence/display/CCRMTDPBeu/MTL+PUBLIC+COMPOUNDS and https://ccrod.cancer.gov/confluence/display/CCRMTDPBeu/Publications+of+MTL+Scientists%2C+2002-2009 and their publications at, http://home.ncifcrf.gov/mtdp/Catalog/catalog.html.

Even the finding of the same or similar compounds can be useful, for example in identifying an abundant source of a previously scarce substance, in establishing chemotaxonomy, or in the identification of new homologues of a bioactive molecule helpful in the pursuit of Structure Activity Relationship (SAR) information. Examples from each of these categories follow:

Jaspamide, (jasplakinolide, CAS 219774-75-1) was such a scarce resource, obtainable in only mg amounts following tedious purification from a sponge. The compound could not be fully investigated as an anticancer lead drug for lack of a supply. The NPSG lab was requested to explore our existing NCI-Frederick Natural Products Repository as a source. Approx. 85 sponge extracts obtained from *Jaspis*, *Auletta*, *Dorypleres*, and other genera from a broad region of the South Pacific where jaspamide had been reported were examined by HPLC. Jaspamide was detectable in a number of extracts which had been obtained from New Guinea, Fiji, the Philippines, and elsewhere, but a single collection identified as a *Dorypleres* sp. which had come from Palau, was very rich in the desired molecule. From the 845 gms of organic solvent soluble extract, 1.3 gms of jaspamide was purified for NCI research [9].

Anticancer screening identified the known fungal-produced compound brefeldin (CAS 20350-15-6) as of interest. Brefeldin A has, of course, been detected from many sources, not all of them fungal [10,11,12]. Fermentation of a number of fungal strains lead to a *Eupenicillium brefeldianum *culture as a moderate producer*,* which was adapted for large-scale liquid fermentation. At the NCI-Frederick fermentation facility, four fermentations at the 12,000 L scale, with purification of the compound by the NPSG, resulted in the production of 4 kg of crystalline brefeldin A for NCI research [13].

Camptothecin (CAS 7689-03-4), a natural product originally isolated from the Asian tree *Camptotheca acuminata *[14], which has been semisynthetically modified into the clinically used anticancer drug topotecan (CAS 123948-87-8), shows bioactivity in many test systems, including a high throughput screen to detect inhibitors of the promising anticancer target HIF-1α. In screening extracts for inhibitors of HIF-1α, an extract obtained from an *Ophiorrhiza trichocarpon *specimen was identified as of interest. Upon dereplication, the extract was found to contain not only camptothecin but additionally, 9-methoxy-, 10-methoxy- and 9,10-methylenedioxy camptothecins [15]. This genus had not previously been reported to contain camptothecin.

A large crude extract library can have value in chemotaxonomic investigations. The marine natural product bryostatin 1 (CAS 83314-01-6), obtained from the bryozoan *Bugula neritina,* is of interest as an anticancer compound having activity through interaction with the diacylglycerol binding site on the cell membrane, the same binding site affected by the irritant and co-carcinogenic phorbol esters which are present in some plants. Bryostatin is present in the marine organism at only a few thousandths of a percent by weight, so tracking its presence during extraction and initial purification via a sensitive bioassay was preferable to chromatographic analysis. At this facility a project to isolate a multi-gram quantity of bryostatin A for research use was initiated [16], and a bioassay measuring displacement of radioactive phorbol dibutyrate (PDBu, CAS 37558-16-0) from rat brain homogenate was developed. Though some members of the plant family Euphorbiaceae are known to produce phorbol esters, there had been no chemotaxonomic survey of the family to determine how widely spread these substances were. To demonstrate the robustness of the PDBu displacement assay when applied to a chemically complex crude fraction, several hundred organic solvent extracts from the Euphorbiaceae, obtained from the NCI-Frederick repository, were run in the displacement assay. When PDBu displacement results were organized according to traditional taxonomy of the Euphorbiaceae, the correspondence was found to be quite close [17,18,19], adding to our knowledge of the distribution of these potentially hazardous natural substances in the environment, and to the chemotaxonomy of the family Euphorbiaceae. Additional investigation would be required to determine the chemotype responsible for the positive PDBu displacement result, or whether a displacement result was produced by a non-phorbol molecule. And at the conclusion of the bryostatin scale-up project, several grams of highly purified bryostatin 1 were produced for drug development studies. 

Serendipitous discoveries are made only because both HTS and a large natural products screening library are available. An example of this came with the observation of an anomalous response in the HTS screen designed to detect compounds which inhibit the HIF-1α protein. An extract demonstrating HIF-1α inhibition would give a decrease in signal from the luciferase reporter engineered into the U251-HRE human glioma cell line, but a small subset of extracts gave a great increase in signal. Upon database mining, this small group of ‘enhancer’ extracts was found to have come from Rubiaceae which had been collected in east Africa and Madagascar. Dereplication of several extracts revealed a common constituent: a known substance, FK228, which had only been reported previously as bacterially-produced [20]. FK228, or FR901228, also known as romidepsin, is a potent histone deacetylase inhibitor (HDAC). Thus the unexpected response was attributable to hyperacetylation, leading to over-expression of the luciferase reporter. By HPLC/MS, a survey of ‘enhancer’ extracts showed the presence of FK228 in all those Rubiaceae extracts giving the high luciferase signal [21]. 

Some particularly interesting stories have developed from the screening program, one of which involved the discovery of a new compound, a new chemotype and description of a new species of plant. A routine specimen of leaves received from Cameroon, West Africa, was identified as *Ancistrocladus abbreviatus*. It was ground, extracted, and the extract made a part of the screening library. In HTS for substances active against the Human Immunodeficiency Virus (HIV), the virus responsible for causing Acquired Immunodeficiency Syndrome (AIDS) in humans, this *Ancistrocladus *extract inhibited cell death of HIV-infected human CEM-SS cells. The compound responsible for this desirable biological response was identified and found to be of a new chemotype of alkaloid, an unusual ‘dimer of dimers’, and given the trivial name of michellamine B, (CAS 137793-81-8), [22], but several subsequent recollections of *A. abbreviatus* obtained from Cameroon failed to contain the same compound. Upon detailed examination of the genus *Ancistrocladus* by plant taxonomists, it was determined that the biologically active molecule was contained in a new species, subsequently described [23]. This rare species of the genus *Ancistrocladus* is localized to a small region of western Cameroon and eastern Nigeria, a significant portion of its range protected in Korup National Park - thus the new species name it received: *A. korupensis.* It is probable, even likely, that this species would never have been described, nor the unusual alkaloid discovered, except for a random collection of the plant and screening of its extract for anti-HIV activity. The NPSG ground and extracted several hundred kg of *Ancistrocladus korupensis *leaves for large scale isolation of michellamine B. But because unacceptable toxicity was observed when michellamine B was administered to dogs, no further development was done.

A collection of *Calophyllum lanigerum* from the Malaysian State of Sarawak, likewise showed activity in the anti-HIV screen, with the bioactive new substance identified and named calanolide A, (CAS 142632-32-4), [24]. When collectors went back to the site to obtain additional plant material, the original source trees had disappeared, a sad example of loss of tropical biodiversity. A chemo-taxonomic survey of a great many trees from the surrounding area failed to find another containing calanolide A. Subsequently, a structurally related molecule having similar biological activity, costatolide, (CAS 63023-58-5) was found which is obtainable in quantity from the latex of a *Calophyllum* spp., [25]. The NPSG developed a procedure for extraction and purification of costatolide which lead to several hundred grams being produced for research. These discoveries contributed to moves by the Sarawak Government to promote conservation measures and the establishment of the Sarawak Biodiversity Center (http://www.sbc.org.my/) which is now conducting in-state investigation of the State’s valuable and diverse biological resources.

HTS in a whole-organism screen designed to uncover compounds useful in the treatment of drug-resistant microbial infections identified many extracts toxic to the test microorganisms, of which two examples deserve mention in this context. An extract from *Aniba panurensis* collected in Guyana showed potent and selective killing of an azole-resistant strain of *Candida albicans*. Following an activity-directed isolation procedure, the toxic substance was isolated, and its structure determined to be a new indolizinium alkaloid (CAS 725265-00-9), [26]. Unfortunately, this molecule does not possess drug-like properties and has low water solubility, so despite its anti-Candida activity, there was no interest in development of this substance as a new drug. 

The same HTS identified an extract from fermentation of an at-that-time unidentified microorganism, later to be determined as a strain of *Aspergillus flavus*, as highly cytotoxic to azole-resistant *C. albicans*. Following activity-guided fractionation, the most fungitoxic substance in the extract was identified as an already known molecule, aspirochlorine (CAS 59978-04-0), but additionally in this extract were trace amounts of two previously unknown aspirochlorine homologues wherein the sulfur bridge contained three or four sulfur atoms, [27].

During the late 1960’s and early 1970’s the DTP supported an anticancer drug discovery program looking for new chemotherapeutic agents in plants. A success from this previous screening program was discovery of the highly effective anticancer drug taxol [28]. Remaining from this program were approximately 6,000 plant collections that had not been extracted. These were ground and extracted by the new protocol given here, and these extracts added into the present screening library. It has not been an infrequent question and implied criticism: “What do you expect to find in 20 year old plants?” In addition to detection of known compounds with new screens, and the resupply of scarce known compounds for research use, new bioactive substances continue to be detected even from old plant specimens. An example is found from an extract prepared from a *Crossosoma bigelovii *specimen collected in Mexico in 1990, and during the intervening years stored dry but not in a temperature-controlled location, which showed activity in an HTS HIF-1α inhibition assay. Activity directed isolation lead to the identification of a new strophanthidin glycoside as being the molecule responsible for HIF-1α inhibitory activity [29].

More importantly than the discovery and re-discovery of bioactive compounds in organic solvent extracts is the exploration of water extracts for new anticancer drugs. Twenty years ago the chromatographic techniques available for the purification of water soluble substances were quite limited, and the spectroscopic equipment and techniques available for solving the larger molecular structures found in water extracts were primitive at best, so most drug discovery organizations made no effort at looking for new drugs in water extracts. Over the intervening years new chromatographic supports (such as C_18_ phase bonded materials suitable for use in 100% water, hydrophilic interaction columns, *etc*.) applicable to resolution of water soluble compounds and new analytical equipment and methods (countercurrent and pH electrophoresis, TOF mass spectrometry, automated Edman degradation sequencing or sequencing by MS, *etc*.) have been developed, overcoming many of the previous deficiencies, and making the search for new drug substances contained in highly polar water soluble mixtures possible. 

With twenty years of experience we can now say that quite a wide range of biologically-active substances have been identified in water extracts. Those chemotypes that would be anticipated, such as sulfated sterols in marine invertebrate extracts, *i.e.* the anti-HIV substance ibisterol sulfate (CAS 148101-50-2), [31], and relatively small macrolide lactones, *i.e.* a compound discovered as a result of anticancer activity in the NCI 60 cell line screen: lobatamide, (CAS 200563-47-9), [32] have been found. But most prominent among the compounds discovered from water-soluble extracts are cyanovirin, [33] produced by a blue-green alga, and griffithsin, [34] produced by a red alga. Both are small proteins, 101 residues for cyanovirin, and 121 for griffithsin, and both are currently being pursued as potential new anti-HIV chemotherapeutics with different modes of action.

An aqueous extract from the sponge *Pipestella candelabra* sp. nov. displayed a cytotoxicity pattern in the 60 cell primary screen which resulted in its selection for testing in the *in vivo* hollow fiber mouse anticancer model, [35]. Interesting *in vivo* biological activity was detected, which resulted in dereplication of this extract. Activity directed isolation lead to the discovery that this sponge extract contains three known bioactive substances, jaspamide (jasplakinolide), hemiasterlin, and brefeldin A, none of which had displayed a comparable 60 cell activity pattern when tested as pure compounds, but in this combination, showed an as-yet unexplained synergy [36].

## 10. Conclusions

We have demonstrated that high throughput extraction of plants, marine specimens and fungal cultures, with precautions taken for preservation of biologically active, chemically sensitive molecules, in support of high throughput screening for anticancer drug discovery, is possible. The diversity of bioactive compounds already discovered in this library of extracts attests to the preservation of the bioactive molecules originally present in specimens by the processing methods presented here. In addition to the traditional organic solvent soluble portion from which most traditionally-used, and presently-used, small molecule drugs have been obtained, an additional diversity of new, biologically active substances has now been shown to be present in water extracts. This large and diverse screening library, containing an unknown number of bioactive molecules, will continue to be re-utilized many times over as the feedstock for new bioassays as they are developed, to probe new molecular targets as they are discovered, and as a challenge for chromatographers and natural products chemists for many years to come. 
